# Quantifying the stochastic component of epigenetic aging

**DOI:** 10.1038/s43587-024-00600-8

**Published:** 2024-05-09

**Authors:** Huige Tong, Varun B. Dwaraka, Qingwen Chen, Qi Luo, Jessica A. Lasky-Su, Ryan Smith, Andrew E. Teschendorff

**Affiliations:** 1 CAS Key Laboratory of Computational Biology, Shanghai Institute of Nutrition and Health, University of Chinese Academy of Sciences, Chinese Academy of Sciences, Shanghai, China; 2 TruDiagnostics, Lexington, KY USA; 3 Channing Division of Network Medicine, Department of Medicine, Brigham and Women’s Hospital and Harvard Medical School, Boston, MA USA

**Keywords:** Computational biology and bioinformatics, Epigenomics, Ageing, Ageing

## Abstract

DNA methylation clocks can accurately estimate chronological age and, to some extent, also biological age, yet the process by which age-associated DNA methylation (DNAm) changes are acquired appears to be quasi-stochastic, raising a fundamental question: how much of an epigenetic clock’s predictive accuracy could be explained by a stochastic process of DNAm change? Here, using DNAm data from sorted immune cells, we build realistic simulation models, subsequently demonstrating in over 22,770 sorted and whole-blood samples from 25 independent cohorts that approximately 66–75% of the accuracy underpinning Horvath’s clock could be driven by a stochastic process. This fraction increases to 90% for the more accurate Zhang’s clock, but is lower (63%) for the PhenoAge clock, suggesting that biological aging is reflected by nonstochastic processes. Confirming this, we demonstrate that Horvath’s age acceleration in males and PhenoAge’s age acceleration in severe coronavirus disease 2019 cases and smokers are not driven by an increased rate of stochastic change but by nonstochastic processes. These results significantly deepen our understanding and interpretation of epigenetic clocks.

## Abstract (web summary)

Tong et al. construct simulations using DNA methylation data to quantify what proportion of the predictive accuracy of epigenetic clocks could be explained by stochastic methylation changes, suggesting that stochasticity contributes more toward the accuracy of chronological rather than biological age predictions.

## Main

Aging at the cellular level is associated not only with genomic abnormalities^1–4^ but also epigenetic ones^5–10^. The development of bead array technologies two decades ago allowed accurate quantification of DNA methylation (DNAm) in whole tissues at thousands of CpGs^9,11^, with early studies indicating that specific age-associated DNAm changes appear to be tissue and cell type independent^12,13^. These initial findings paved the way for the development of epigenetic clocks, defined as multivariate linear predictors of chronological age, capable of estimating chronological age in multiple tissue types with a remarkable degree of accuracy (for example, Horvath’s clock displays a median absolute error (MAE) of ±3–5 years)^14–18^. In this epigenetic clock framework, samples displaying abnormally large deviations from their true ages were hypothesized to age faster or slower, yielding molecular estimates of biological age^14^, with subsequent studies confirming that DNAm-based biological age estimates can be informative of future disease risk and mortality^19,20^. Intuitively, the more accurate an epigenetic clock is in predicting chronological age (for example, Zhang’s clock^21^), the less informative it can be of biological age. Conversely, clocks that are more informative of biological age (for example, the PhenoAge^22^ or GrimAge clocks^23^) are less predictive of chronological age^18,24^.

In parallel to the development of epigenetic clocks, numerous studies have analyzed the spatial and systems-level patterns of age-associated epigenetic changes, including DNAm and chromatin^12,13,25–34^. These studies have unequivocally shown that specific genomic regions are more likely to acquire age-associated DNAm changes than others, with CpGs marked by the polycomb-repressive complex-2 (PRC2) or bivalent marks in stem cells being one clear example^12,13,30,35^. Moreover, studies have shown that genomic regions that start out unmethylated in a suitably defined ground state (for example, promoter CpG islands in fetal tissue) tend to gain DNAm with age^28^, while regions that are generally methylated (for example, open sea and exon bodies) or partially methylated (partially methylated domains) tend to lose DNAm^25,32^. This gradual erosion of the normal DNAm landscape where initially well-demarcated boundaries between methylated and unmethylated regions become gradually blurred also appears to be largely stochastic in the sense that neighboring CpGs do not necessarily change synchronously or by the same amount^33,34,36^. Indeed, the recent study by Tarkhov et al. concluded that most age-associated DNAm changes are devoid of nonstochastic coregulatory patterns^33^. Thus, overall, the pattern of age-associated DNAm change in the genome appears to be ‘quasi-stochastic’ in the sense that specific regions are more likely to acquire DNAm changes, but that once restricted to these regions, the patterns appear more random. When viewed across the whole genome, the DNAm distribution becomes more stochastic or uniform with age, thus defining a state of higher statistical entropy. From the perspective of single CpGs that begin as either unmethylated or fully methylated, their DNAm values generally approach more intermediate values reflecting a higher uncertainty or entropy in the DNAm distribution defined over single cells^15,33^.

While it may be counterintuitive that a largely stochastic process of age-associated molecular change could allow for the construction of an accurate, aka deterministic, predictor of chronological age, this is in fact guaranteed by the intrinsic linearity in which any counter of DNA alterations, measured relative to a well-defined ground state, changes within a predefined unit of time^33,34^ (Methods). However, this insight also begs a fundamental question in aging, namely, how much of the accuracy displayed by an epigenetic clock such as Horvath’s can be attributed to an underlying pure stochastic process? In this Analysis, we use state-of-the-art methodology and a large collection of independent DNAm datasets to rigorously address this question, demonstrating that the fraction of an epigenetic clock’s accuracy that could be explained by a pure stochastic process increases with the clock’s predictive ability. This is consistent with the notion that biological aging, as measured by a clock such as PhenoAge, is driven by nonstochastic processes^37^ and not merely by an increased rate of stochastic change.

## Results

### Strategy to quantify stochasticity of epigenetic aging

**Fig. 1:**
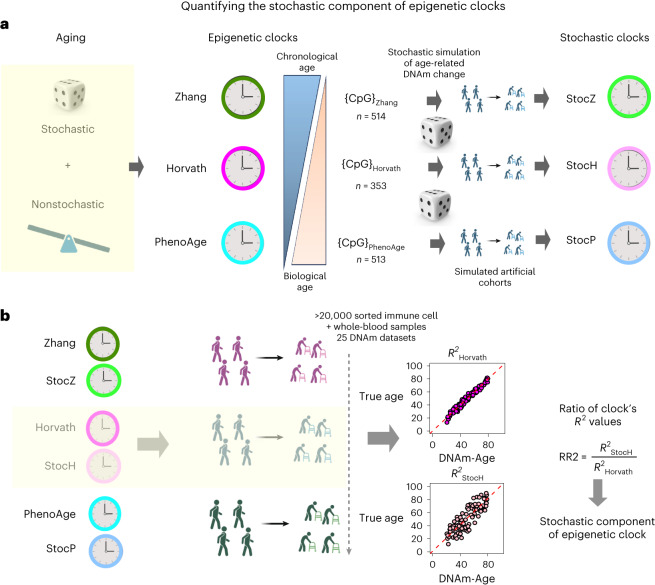
The overall strategy to quantify the stochastic component of epigenetic clocks. **a**, The original epigenetic clocks are constructed from real DNAm data describing a real aging process that includes both stochastic and nonstochastic elements. The key idea is to simulate a realistic stochastic process of DNAm change accrual at the same CpG sites that make up the original clocks. In effect, this ‘replaces’ the real aging process with a pure stochastic one at the same sites. With the simulation model in place, one can then generate artificial cohorts from which machine learning predictor of age can be derived, defining the ‘stochastic clock’ analogs. **b**, We then apply the original and stochastic clocks to a large collection of DNAm datasets representing both sorted immune cells (to gauge the effect of CTH) and whole-blood tissue. The ratio of *R*^2^ values between the stochastic clock and its original counterpart provides a direct quantification of the stochastic component of that clock. Created with Biorender.com.

We reasoned that a way to quantify the stochastic component of epigenetic aging is to take the CpGs that make up current epigenetic clocks and to simulate a stochastic process of DNAm-change accrual at these sites, using only information about their effect sizes and directionality of change (Fig. 1a). Once the stochastic simulation model is specified, artificial DNAm datasets can be generated. From these simulated datasets, machine learning predictors of chronological age can then be derived. As they are derived from data generated by a pure stochastic process, we call these clocks ‘stochastic’ (Fig. 1a). In contrast, the original epigenetic clocks are derived from real DNAm datasets describing a real aging process that is thought to include both stochastic and nonstochastic elements. Importantly, for any given epigenetic clock, the original and stochastic clocks are defined over the exact same CpGs. To quantify the stochastic component of an epigenetic clock, we apply the original and its stochastic clock counterpart to each one of a large collection of sorted immune cell and whole-blood DNAm datasets (Fig. 1b). For each of these cohorts, one can then estimate the stochastic component as the ratio of the two clock’s *R*^2^ values (abbreviated as RR2), where the *R*^2^ of a given clock quantifies the fraction of age variation explained by that clock (Fig. 1b). In more detail, RR2 is defined as the ratio of the stochastic clock’s *R*^2^ value to the *R*^2^ value of the original clock (Fig. 1b). Biologically, this ratio measures how much of a given clock’s accuracy (that is, the *R*^2^ or age variation explained) could be driven by a pure stochastic process defined over the same set of CpGs. Taking the ratio is important because the actual *R*^2^ value attained by a clock in any given human cohort may be influenced by study-specific factors such as age range, normalization and batch effects, environmental exposures or comorbid conditions. As the two clocks being compared are defined over the same set of CpGs, these study/cohort-specific factors will influence both clocks equally, so that taking the ratio of the *R*^2^ value will naturally adjust for such study/cohort-specific biases. By using a fairly large number of datasets, robustness of these RR2 estimates can be assessed.

### Simulating stochastic age-associated DNAm changes

**Extended Data Fig. 1:**
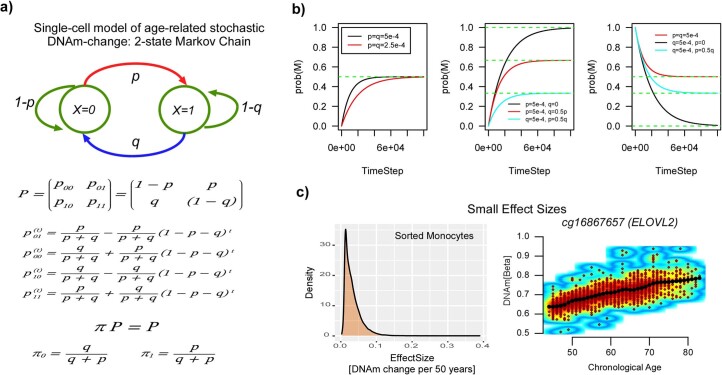
Single-cell stochastic model of DNAm change with age. **a)** Depiction of a 2-state Markov Chain model with *X* = *0* representing the unmethylated state of one CpG, *X* = *1* representing it’s methylated state. *P* is the stochastic matrix so that row-entries add to 1. *π* represents the invariant steady-state probability distribution of finding a CpG in one cell unmethylated or methylated. The solution to the dynamics specified by the Markov-Chain as well as the steady-state values are given. **b)** Examples of how the probability of finding a CpG methylated in one cell changes with timestep for different scenarios specified by *p* (the probability of methylation), *q* (the probability of demethylation) and initial conditions. **c)** Empirically estimated effect sizes for real age-associated CpGs, as derived from a large 450k DNAm dataset of 1202 monocytes. Right scatterplot depicts the top-ranked CpG displaying the largest effect size.

To build a realistic simulation model describing stochastic DNAm changes with age, we begin with a single-cell model at one CpG site. We used a simple two-state model to simulate binary DNAm switches within a single cell (Methods and Extended Data Fig. 1a). This model allows estimation of the probability of a CpG being methylated at any time point and predicts that for an initially unmethylated (or methylated) site, the probability of methylation increase (or decrease) changes linearly with time, until it enters a nonlinear regime close to the steady-state value (Methods and Extended Data Fig. 1b). From this single-cell model, the expected methylation fraction (DNAm beta-value) in a cell population can be computed and shown to also be a linear function of time, unless DNAm values are close to the steady state (Methods). We next used Illumina 450k DNAm data of sorted monocyte samples from 1,202 donors spanning a wide age range (minimum age of 44 years, maximum age of 83 years and mean age of 60 ± 9 years) (MESA study^38^) to demonstrate that, even in a purified cell population, typical effect sizes of age-related DNAm change are very small (that is, <5% over 50 years; Extended Data Fig. 1c). This small effect size means that only a very small fraction of cells in a cell population display binary DNAm changes. Thus, both theoretical considerations, as well as empirical observation, justify using a linear approximation for our stochastic simulation model (Methods).

**Fig. 2:**
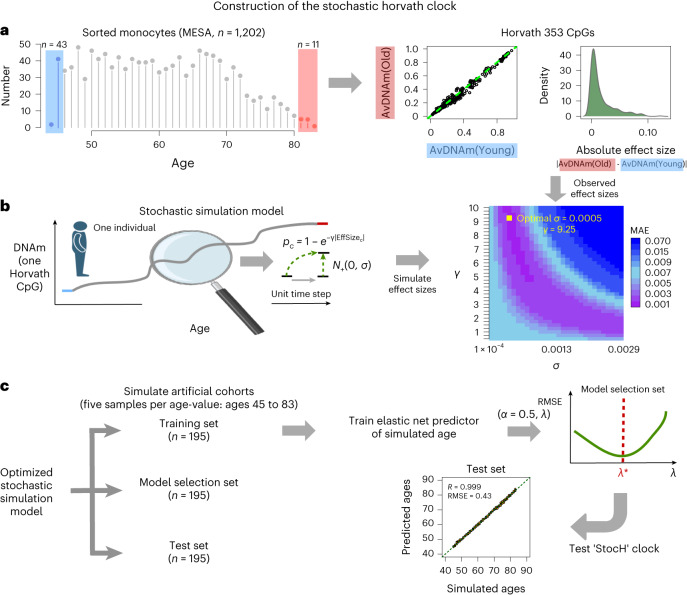
Construction of the StocH clock. **a**, Left: the age distribution of the 1,202 sorted monocyte samples from the MESA study. The shaded blue and red regions highlight the youngest and oldest samples used in the simulation, respectively. Middle: the average DNAm over the youngest (AvDNAm(Young)) and oldest (AvDNAm(Old)) samples for each of the 353 Horvath Clock CpGs. Right: the corresponding density of absolute effect sizes defined as the magnitude of the DNAm difference between youngest and oldest samples. **b**, The stochastic simulation of one CpG in one individual of a given age, which starts out from the average DNAm in the youngest samples and subsequently adds a stochastic deviate for each unit time step. The probability per time step that a CpG is altered is given by a decaying exponential with exponent determined by the observed absolute effect size of the CpG and a CpG-independent parameter, *γ*, that controls the overall probability of CpGs changing. The direction of the DNAm change is dictated by the directionality of the observed effect size, with the magnitude determined by the standard deviation, *σ*, of a signed Gaussian distribution, as indicated. Of note, the simulation model adds Gaussian deviates to the quantiles of an inverse normal distribution. The model is simulated to generate effect sizes for each of the 353 CpGs, which is then compared with the observed distribution to identify the optimal (*λ* and *σ*) parameters minimizing the MAE between simulated and observed values. **c**, To build the StocH clock, we then use the simulation model with the optimal (*λ* and *σ*) parameter values to generate three artificial cohorts of 195 samples each. There are 195 samples because we simulate five samples per age value, with ages ranging between 45 and 83 years; that is, a total of 39 distinct age values. One cohort is used to train elastic net regression models with $\alpha =0.5$, and for varying penalty parameter values, *λ*. These models are then evaluated in the model selection set to select the model that optimizes the root mean square error (RMSE). This optimal model is then evaluated in the test set. Created with Biorender.com.

**Extended Data Fig. 2:**
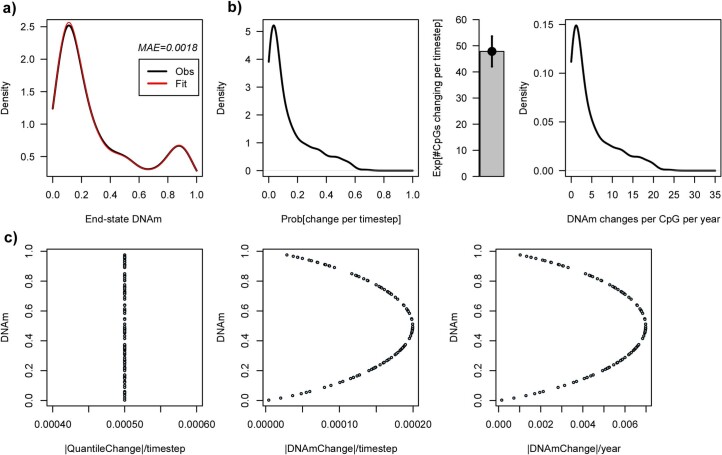
Interpretation of optimal parameter values. **a)** Density plots of the observed (black) and estimated (red) end-state DNAm-values of the 353 Horvath Clock CpGs, with the median absolute error (MAE) given in the plot. **b)** Left: Density plot of the estimated probability of change per unit time step for the 353 Horvath CpGs. Middle: Barplot gives the estimated average number of CpGs (+/- standard deviation) that change per timestep. Mean and standard deviation are analytical estimates from a binomial distribution with n = 353. Right: Density plot of the estimated number of DNAm changes a CpG undergoes per year for all 353 CpGs. **c)** Left: the optimal standard deviation change in the normal quantile basis (x-axis) was estimated to be 5e-4, which is the same irrespective of the actual DNAm-value (y-axis), reflecting the desire to have homoscedastic changes. Middle: corresponding plot when considering the change in the DNAm-value basis, displaying the natural heteroscedasticity of beta-values, as required. Right: as Middle, but now plotting the x-axis on the scale of DNAm change per year, that is the maximum DNAm change per year amounts to about 0.8% (that is 0.008) in units of beta-values.

Since the ultimate aim is to build an epigenetic clock from a stochastic age-related process, we next extended the previous model to incorporate realistic effect sizes, focusing initially on the 353 CpGs that make up Horvath’s clock. Although it is now well recognized that other CpG combinations could be used to build equally accurate age predictors^17^, here we only focus on the original 353 CpGs since we wish to directly compare to the original Horvath clock. To avoid confounding by cell type heterogeneity (CTH) we estimated their age-associated effect sizes from the sorted monocyte MESA samples^38^, but now using only the youngest (age <46 years, *n* = 43) and oldest (age >80 years, *n* = 11) samples (Methods and Fig. 2a). In our simulation model, the absolute effect size of each CpG determines the relative probability of that CpG being altered per unit time step, with a global parameter γ determining the overall probability of undergoing a DNAm change (Methods and Fig. 2b). The magnitude of DNAm change per unit time step is controlled by the standard deviation (*σ*) of a Gaussian distribution, where the normal deviates are added stochastically to the relevant CpG using the inverse normal quantiles of their DNAm beta-values (Methods and Fig. 2b). Thus, our model is parsimonious in only including two parameters (*γ* and *σ*) that we tune so that the simulated end-state DNAm value distribution over the 353 CpGs is as similar as possible to the observed one (Fig. 2c and Extended Data Fig. 2). While the time step unit is arbitrary, its scale influences the optimal (*γ* and *σ*) values. To aid biological intuition, and since we are dealing with an immune cell type for which the annual intrinsic number of stem cell divisions is approximately 35 (see, for example, ref. ^39^), we run the simulation model for a total of 35 × 37 = 1,295 time steps, corresponding to 1,295 ‘stem cell divisions’ over the course of 37 years (average age of old subjects, 82 years and average age of young subjects, 45 years). We note that the model does not require the process associated with the DNAm changes to be cell division but doing so helps anchor the interpretation of the actual parameter values. The inferred optimal parameter values (*γ* = 9.25 and *σ* = 0.0005) gave an excellent fit (MAE of 0.0018) to the observed end-state DNAm values (Fig. 2b and Extended Data Fig. 2a). These parameter values imply that, on average, 48 of 353 CpGs (that is, 14%) change at every time step (Extended Data Fig. 2b) and that the magnitude of average DNAm change per CpG per year is less than 0.1% (that is, a 1% DNAm change over a decade), with the actual magnitude of DNAm change displaying the characteristic heteroscedasticity of beta-values, as required (Extended Data Fig. 2c).

### Construction and validation of the StocH clock

Having built and tuned a model of stochastic DNAm change at the 353 Horvath CpG sites, we next used this model to simulate an artificial cohort, with the aim to then derive a ‘stochastic’ analog of Horvath’s clock, that is an elastic net regression predictor of chronological age (Methods and Fig. 2c). We simulated a total of 39 ages, spanning the range 45–83 years old, assuming 35 ‘stem-cell divisions’ (that is, time steps) per year and with five independent samples per age value (Methods), resulting in an artificial cohort of 195 samples. Of note, the initial DNAm profile (that is, the profile at age 45 years) was always chosen randomly from the pool of youngest samples (*n* = 41). Elastic net regression models^40^ for a range of different penalty parameter values were then trained and the best-performing model selected using an independently generated artificial DNAm dataset of 195 samples (Fig. 2c). We call the resulting optimal elastic net regression model the ‘stochastic Horvath clock’ or ‘StocH clock’ for brevity. A third independently generated artificial DNAm dataset was then used to confirm that the optimal model can predict the simulated age with high accuracy (Fig. 2c).

### StocH clock predicts age in sorted cells

**Fig. 3:**
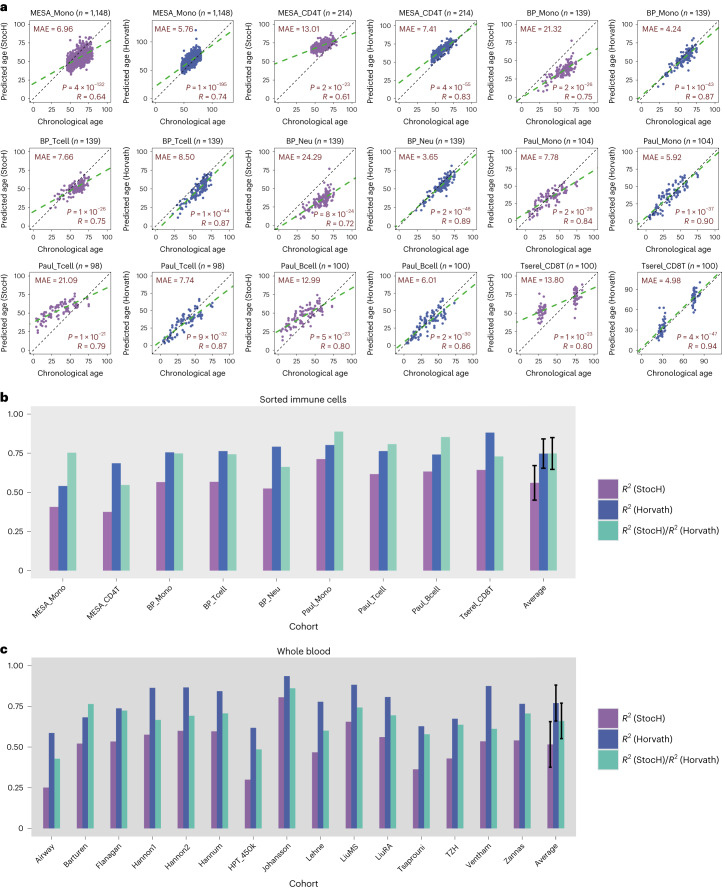
Quantification of stochastic component of Horvath’s clock. **a**, Scatter plots of predicted age versus chronological (true) age for the StocH clock (purple) and Horvath’s clock (slate blue) in various sorted immune cell datasets as indicated. The number of sorted samples in dataset is indicated at the top alongside the name of the cohort/study. In addition, we provide the MAE, *R*-value and corresponding nominal correlation-test two-tailed *P* value in each plot. **b**, A bar plot comparing *R*^2^ values of the StocH clock and Horvath’s clock in each of the datasets. In addition, we also depict the ratio of the *R*^2^ value from StocH to the *R*^2^ value from Horvath’s clock. The sample sizes of sorted immune cell datasets are as follows: MESA_Mono (*n* = 1,148), MESA_CD4T (*n* = 214), BP_Mono (*n* = 139), BP_Tcell (*n* = 139), BP_Neu (*n* = 139), Paul_Mono (*n* = 104), Paul_Tcell (*n* = 98), Paul_Bcell (*n* = 100) and Tserel_CD8T (*n* = 100). The last set of bars displays the average and standard deviation over all sorted immune cell datasets. **c**, As in **b**, but for StocH and Horvath’s clock on 15 whole-blood cohorts. The sample sizes of whole-blood datasets are as follows: Airway (*n* = 1,032), Barturen (*n* = 574), Flanagan (*n* = 184), Hannon1 (*n* = 636), Hannon2 (*n* = 665), Hannum (*n* = 656), HPT_450k (*n* = 418), Johansson (*n* = 729), Lehne (n = 2,707), LiuMS (*n* = 279), LiuRA (*n* = 689), Tsaprouni (*n* = 464), TZH (*n* = 705), Ventham (*n* = 380) and Zannas (*n* = 422).

As the StocH clock was trained using information from only the youngest and oldest monocyte MESA samples, it is legitimate to ask if the StocH clock can predict the chronological age of all other MESA samples (*n* = 1,148) not used in building the clock. On these samples, the StocH clock attained an *R*-value of 0.64 (*P* < 10 × 1^−100^) and a MAE of 6.96, comparable to Horvath’s clock itself (*R* = 0.74, *P* < 10 × 1^−100^ and MAE of 5.76) (Fig. 3a). However, when we assessed both clocks in the 214 sorted CD4^+^ T cell samples from the same MESA study, Horvath’s clock performed significantly better with respect to the MAE, although the StocH clock remained predictive of chronological age (*R* = 0.61 and *P* < 10 × 1^−20^, Fig. 3a). We verified, using a modified simulation model (Methods), that the increase in MAE displayed by the StocH clock in the CD4^+^ T cells is not due to any dependency of the StocH clock on ground-state DNAm values that are characteristic of monocytes (Supplementary Fig. 1). Indeed, when we applied the StocH clock to the Illumina 450k DNAm data of sorted monocytes, neutrophils and T cells from BLUEPRINT^41^, the MAE displayed in T cells was much better than in monocytes (Fig. 3a), suggesting no obvious dependence on the ground-state DNAm of the actual cell type.

### Quantifying the stochastic component of Horvath’s clock

**Extended Data Fig. 3:**
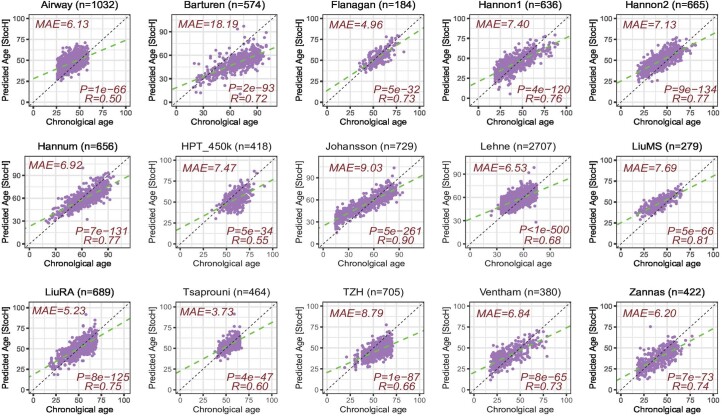
Stochastic Horvath (StocH) clock predicts chronological age in whole blood cohorts. Scatterplots of the true age (x-axis) vs predicted age (y-axis) for the StocH clock in 15 whole blood cohorts. Number of samples in each cohort is indicated above each plot. Median Absolute Error (MAE), R-value and associated nominal correlation-test two-tailed P-value are given.

Given that our StocH clock is made up of exactly the same Horvath clock CpGs, its accuracy of prediction relative to Horvath’s clock provides a quantification of the stochastic component underlying Horvath’s clock. Indeed, as reasoned earlier, RR2 (that is, the *R*^2^ of the StocH clock divided by the *R*^2^ of Horvath’s clock; Fig. 1b) would be a suitable measure to directly quantify the stochastic component of Horvath’s clock, since the ratio automatically adjusts for any intrinsic study-specific biases (Methods). Supporting this, we note that across all sorted immune cell datasets analyzed here, Horvath’s clock always displayed better prediction performance than its stochastic analog, with the RR2 being approximately 0.75 ± 0.10 (Fig. 3b). To further test this, we assembled 15 whole-blood cohorts, encompassing a total of 10,540 samples (Methods). Once again, the StocH clock was able to predict chronological age in all cohorts, often with a remarkably high degree of accuracy, even in terms of MAE (Extended Data Fig. 3), but never outperforming Horvath’s clock (Fig. 3c). Averaged over the 15 independent cohorts, the RR2 was 0.66 ± 0.11 (Fig. 3c), which is within one standard deviation of the estimate obtained in sorted immune cell datasets. Put together, these results indicate that approximately 66–75% of the relative accuracy underlying Horvath’s clock could be driven by an underlying stochastic process.

### Stochasticity underpins accuracy of chronological age prediction

**Fig. 4:**
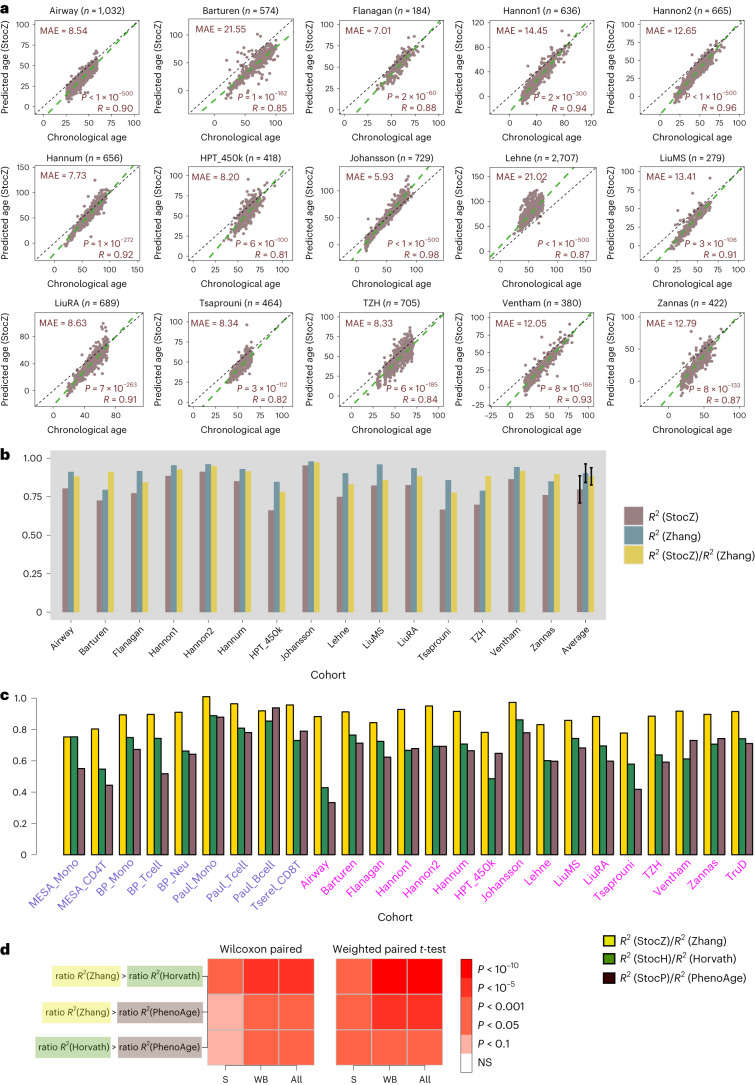
Stochastic component increases with accuracy of clock. **a**, Scatter plots of predicted age versus chronological (true) age for the stochastic Zhang clock (StocZ clock) in each of 15 whole-blood datasets. The number of samples in the dataset is indicated at the top alongside the name of the cohort. In addition we provide the MAE, *R*-value and corresponding nominal correlation-test two-tailed *P* value in each plot. **b**, A bar plot comparing *R*^2^ values of the StocZ clock and Zhang’s clock in each of the 15 whole-blood datasets. In addition, we also depict the ratio of the *R*^2^ value from StocZ to the *R*^2^ value from Zhang’s clock. The last set of bars displays the average and standard deviation over the 15 datasets. The sample size of each cohort is given in **a**. **c**, A comparison of the ratio of *R*^2^ values among Zhang, Horvath and PhenoAge clocks across all sorted and whole-blood datasets. The sample size of the sorted datasets are given in Fig. 3 legend. **d**, Heat maps displaying meta-analysis *P* values comparing the ratio of *R*^2^ values across the sorted datasets (S), the whole-blood datasets (WB) and all together (All). We provide two sets of *P* values, one derived from a one-tailed paired Wilcoxon rank sum test over cohorts and another derived by running a weighted linear regression model of the ratios against clock with the cohort as a covariate and using the sample sizes of the cohorts as weights. In the latter case, the *P* value is two tailed.

**Extended Data Fig. 4:**
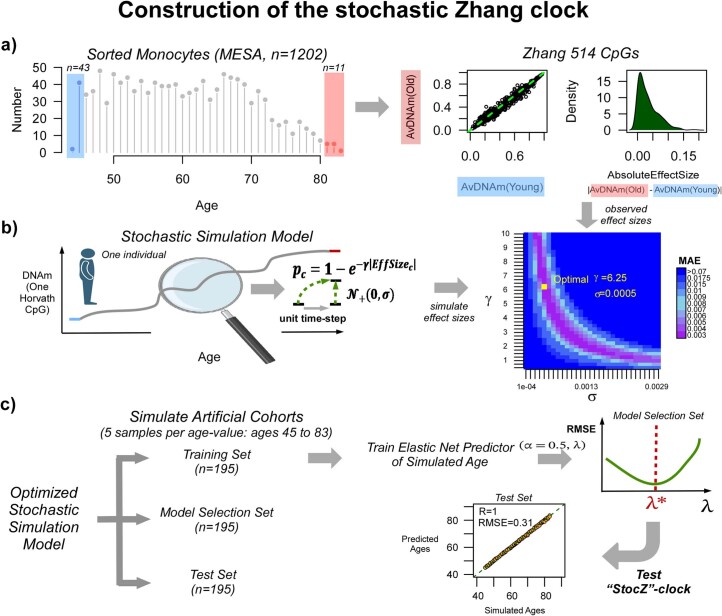
Construction of the Stochastic Zhang (StocZ) clock. **a)** Left: age distribution of the 1202 sorted monocyte samples from the MESA study, for which Illumina 450k DNAm profiles have been generated. The shaded blue and red regions highlight the youngest and oldest samples that are used in the simulation model. Middle panel displays the average DNAm over the youngest and oldest samples for each of the 514 Zhang Clock CpGs and right panel displays the corresponding density of absolute effect sizes defined as the magnitude of the DNAm difference between youngest and oldest samples. **b)** The stochastic simulation model of one CpG in one individual of a given age, starts out from the average DNAm in the youngest samples, and subsequently adds a stochastic deviate for each unit time-step. The probability per time-step that a CpG is altered is given by an exponential decaying function with the exponent determined by the observed absolute effect size of the CpG and a CpG-independent parameter, γ, that controls the overall probability of CpGs changing. The direction of the DNAm change is dictated by the directionality of the observed effect size, with the magnitude determined by the standard deviation, σ, of a signed Gaussian distribution, as indicated. Of note, the simulation model adds Gaussian deviates to the quantiles of an inverse Normal distribution, where the DNAm-values are interpreted as probabilities of DNAm. The model is simulated to generate effect sizes for each of the 514 Zhang clock CpGs, which is then compared to the observed distribution to identify the optimal (λ,σ) parameters that minimize the median absolute error (MAE) between simulated and observed values. c) To build the stochastic Zhang clock, we then use the simulation model with the optimal (λ,σ) parameter values to generate 3 artificial cohorts of 195 samples each. There are 195 samples, because we simulate 5 samples per age-value with ages ranging between 45 and 83 that is a total of 39 distinct age-values. One cohort is used to train Elastic Net Regression models with α = 0.5, and for varying penalty parameter values λ. These models are then evaluated in the model selection set to select the model that optimizes the root mean square error (RMSE). This optimal model is then evaluated in the test set.

**Extended Data Fig. 5:**
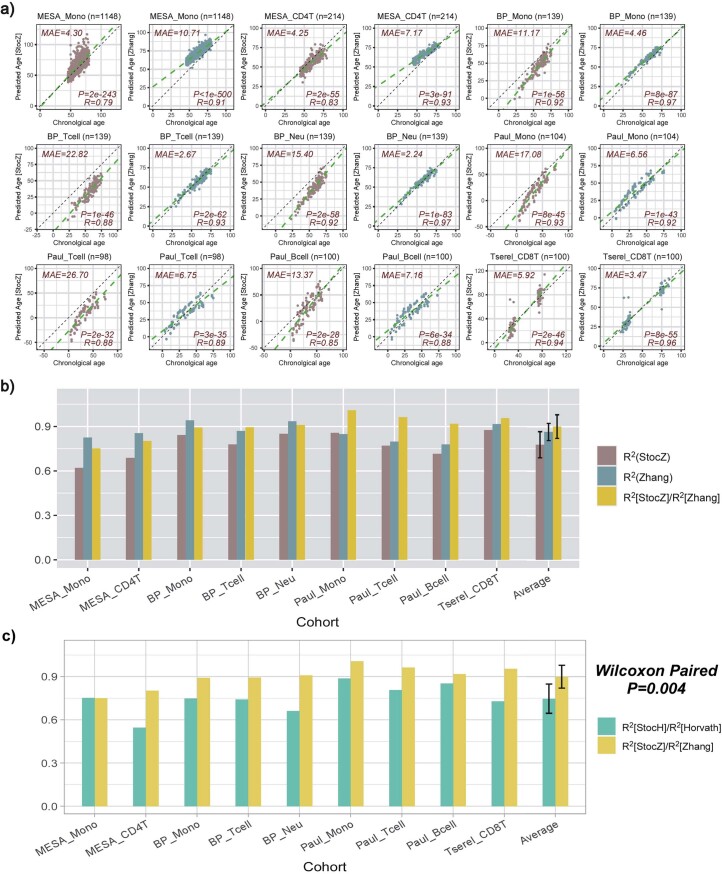
Quantification of the stochastic component of Zhang’s clock in sorted immune cell sets. **a)** Scatterplots of predicted age vs true age for the StocZ-clock (brown) and Zhang’s clock (green) in sorted immune cell datasets. Number of sorted samples in dataset is indicated at the top of the panel alongside the name of the cohort/study. In addition, we provide the median absolute error (MAE), R-value and corresponding nominal correlation-test two-tailed P-value. **b)** Barplot comparing R^2^ values of the StocZ-clock and Zhang’s clock in each of the datasets. We also depict the ratio of the R^2^ value from StocZ to the R^2^ value from Zhang’s clock. Sample sizes are: MESA_Mono (n = 1148), MESA_CD4T (n = 214), BP_Mono (n = 139), BP_Tcell (n = 139), BP_Neu (n = 139), Paul_Mono (n = 104), Paul_Tcell (n = 98), Paul_Bcell (n = 100), Tserel_CD8T (n = 100).The last set of bars displays the average and standard deviation over all sorted immune cell datasets. **c)** Comparison of the ratio of R^2^ values between Horvath and Zhang CpGs. P-value is from a one-tailed paired Wilcoxon rank sum test comparing the ratio of R^2^ values. Sample sizes as in b). The last set of bars displays the average and standard deviation over all sorted immune cell datasets.

Next, we repeated the previous procedure, but now building the stochastic clock from the 514 CpGs that make up Zhang’s clock^21^ (Fig. 1 and Extended Data Fig. 4). Since Zhang’s clock is a more accurate predictor of chronological age and consequently less predictive of biological age^21^, we reasoned that this analysis may shed insight into whether stochastic processes could underpin biological aging. We systematically tested the stochastic analog of Zhang’s clock, the StocZ clock, in the sorted immune cell and whole blood datasets (Fig. 4a,b and Extended Data Fig. 5a,b). This revealed a striking pattern, with the stochastic clock describing a much higher fraction of the epigenetic clock’s accuracy in the case of Zhang’s clock compared with Horvath’s (Fig. 4c and Extended Data Fig. 5c). For instance, on the sorted immune cell datasets, StocZ and Zhang’s clock achieved an average *R*^2^ value of 0.78 ± 0.09 and 0.86 ± 0.06, respectively, with the average RR2 values being 0.90 ± 0.08 (Extended Data Fig. 5b,c). This is much higher than the ratio 0.75 ± 0.10 displayed by the StocH and Horvath clocks. Statistical significance that RR2 values are higher for Zhang’s CpGs compared with Horvath was confirmed using two different statistical tests, including a weighted test that takes cohort size into account (Fig. 4d). Of note, while the above analyses in whole blood included mostly healthy samples, the results remained unchanged upon restriction to only healthy nonsmokers without comorbid conditions (Supplementary Figs. 2 and 3).

**Extended Data Fig. 6:**
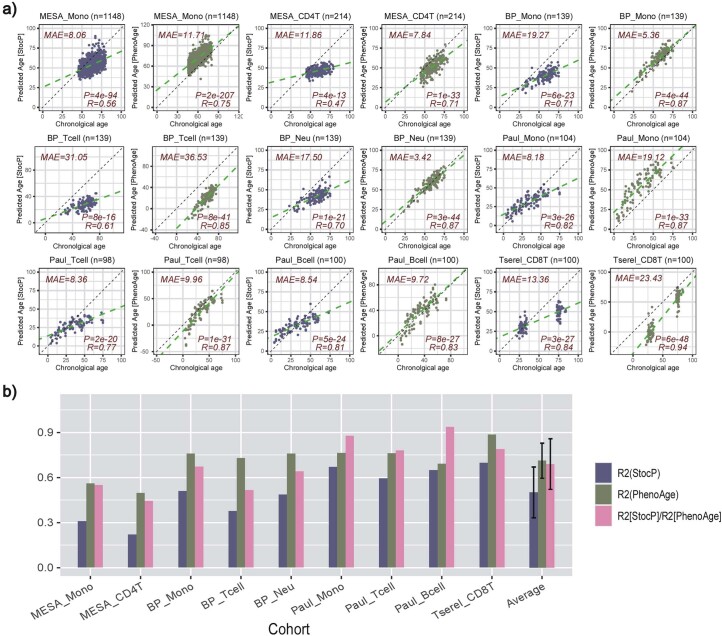
Quantifying stochastic component of PhenoAge-clock in sorted immune cell datasets. **a)** Scatterplots of predicted age vs chronological (true) age for the stochastic PhenoAge-clock (StocP) in sorted immune-cell datasets. Number of samples in dataset is indicated at the top of the panel alongside the name of the cohort/study. In addition, we provide the median absolute error (MAE), R-value and corresponding nominal correlation-test two-tailed P-value. **b)** R^2^ values for StocP, PhenoAge clock and their ratio across all datasets. Sample sizes are: MESA_Mono (n = 1148), MESA_CD4T (n = 214), BP_Mono (n = 139), BP_Tcell (n = 139), BP_Neu (n = 139), Paul_Mono (n = 104), Paul_Tcell (n = 98), Paul_Bcell (n = 100), Tserel_CD8T (n = 100). The last set of bars displays the average and standard deviation over all sorted immune cell datasets.

**Extended Data Fig. 7:**
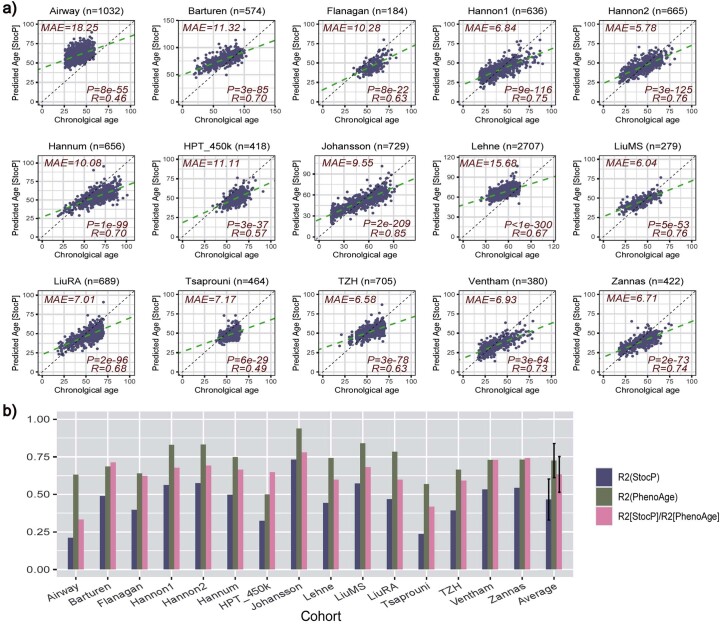
Quantifying stochastic component of PhenoAge-clock in whole blood datasets. **a)** Scatterplots of predicted age vs chronological (true) age for the stochastic PhenoAge-clock (StocP) in whole blood datasets. Number of samples in dataset is indicated at the top of the panel alongside the name of the cohort/study. In addition, we provide the median absolute error (MAE), R-value and corresponding nominal correlation-test two-tailed P-value. **b)** R^2^ values for StocP, PhenoAge clock and their ratio across all datasets. Sample sizes are: Airway (n = 1032), Barturen (n = 574), Flanagan (n = 184), Hannon1 (n = 636), Hannon2 (n = 665), Hannum (n = 656), HPT_450k (n = 418), Johansson (n = 729), Lehne (n = 2707), LiuMS (n = 279), LiuRA (n = 689), Tsaprouni (n = 464), TZH (n = 705), Ventham (n = 380), Zannas (n = 422). The last set of bars displays the average and standard deviation over all whole blood datasets.

**Extended Data Fig. 8:**
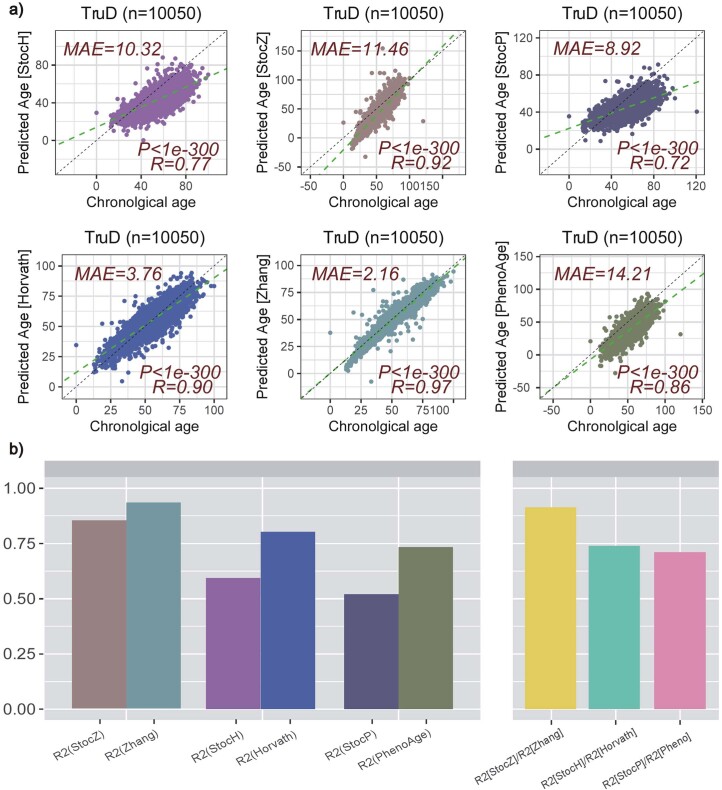
Quantification of stochastic aging component in TruD cohort. **a)** Scatterplot of predicted age vs chronological age for the 3 stochastic clocks (StocH, StocZ and StocP, top row) and their original clocks (Horvath, Zhang and PhenoAge, bottom row). In each case, we provide the MAE, R and nominal correlation-test two-tailed P-value. **b)** Barplot comparing the R^2^ values of the 6 clocks (left) and the ratio of R^2^ values (right).

All these results strongly suggest that, the more accurate an epigenetic clock is in predicting chronological age, the more it could be driven by an underlying stochastic process. Consequently, clocks that are less predictive of chronological age but that are better at predicting biological age are more likely to reflect nonstochastic processes. To test this, we repeated the above analysis but now for the 513 CpGs that make up Levine’s PhenoAge clock^22^ (Fig. 1), building a stochastic clock analog (StocP) and subsequently computing its, as well as PhenoAge’s clock, *R*^2^ values in the sorted immune cell and whole-blood datasets (Extended Data Figs. 6 and 7). To increase power, we added an additional cohort of 10,050 whole-blood samples from mostly healthy individuals (TruD-cohort; Methods and Extended Data Fig. 8)^42^. Confirming our hypothesis, the ratio of *R*^2^ values was significantly lower for StocP/PhenoAge compared with StocZ/Zhang (paired Wilcoxon, *P* < 10^−7^ and weighted paired *t*-test, *P* < 10^−12^) and StocH/Horvath (paired Wilcoxon, *P* = 0.017 and weighted paired *t*-test, *P* < 0.005) (Fig. 4c,d). Thus, these data point toward stochasticity underpinning the accuracy of epigenetic clocks.

**Extended Data Fig. 9:**
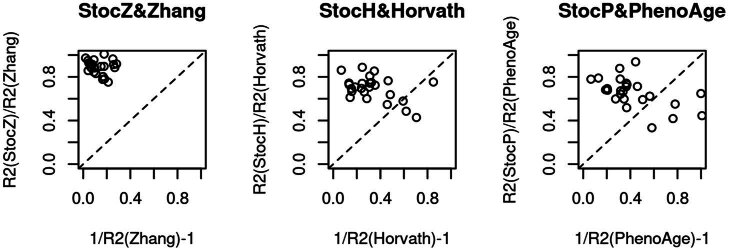
Stochastic clocks do not describe the unexplained age-variation of epigenetic clocks. Scatterplot of RR2 measures (y-axis) against 1/R^2^ – 1 (x-axis) for each clock-type, with the line of proportionality shown as a dashed line.

To further stress this important insight, we next show that an alternative assumption or hypothesis, namely that stochastic clocks describe the age variation not explained by a given clock, is inconsistent with empirical observation. Indeed, according to this alternative hypothesis, *R*^2^(StocClock) ~ 1 − *R*^2^(Clock), which would imply that RR2 ~ 1/*R*^2^ − 1. However, plotting RR2 against 1/*R*^2^ − 1 revealed that data points for each clock type and cohort clustered away from the line of proportionality, with no evidence of a positive correlation (Extended Data Fig. 9).

### Decreased accuracy for clocks built from other CpGs

The StocH, StocZ and StocP clocks were built from the corresponding CpGs that make up Horvath, Zhang and PhenoAge clocks, respectively, and as such, these stochastic clocks indirectly use information gleaned from large numbers of datasets. Indeed, given how Horvath and Zhang clock CpGs were derived, these loci are clearly optimized for linear prediction of chronological age, although it is important to stress here that their selection is naive to the underlying nature of the biological processes that give rise to age-related DNAm changes. Consequently, if we were to build a stochastic clock from age-related CpGs derived from only one study, the predictive performance of a corresponding stochastic clock should drop significantly. To test this, we built a stochastic clock from CpGs undergoing the biggest DNAm differences between the young and old monocyte samples from the MESA study (Supplementary Fig. 4a,b). In this instance, however, we built two clocks, one where model selection (that is, selection of penalty parameter) was done using a separate simulated dataset (the StocF clock, Supplementary Fig. 4c) and another quasi-stochastic clock were the optimal penalty parameter was chosen using 50% of the MESA samples (*n* = 574) (the StocQ clock) that were not used in the CpG selection procedure (Supplementary Fig. 4d). Confirming our hypothesis, these stochastic clocks could not predict chronological age as well as, for example, StocH or StocZ (Supplementary Fig. 4e). Thus, the relatively high accuracy of StocH and StocZ in predicting chronological age hinges on the specific CpGs that make up the original Horvath and Zhang clocks, suggesting that their selection implicitly finds CpGs that are more likely to undergo stochastic DNAm changes with age.

### Age acceleration not driven by an increased rate of stochastic change

**Fig. 5:**
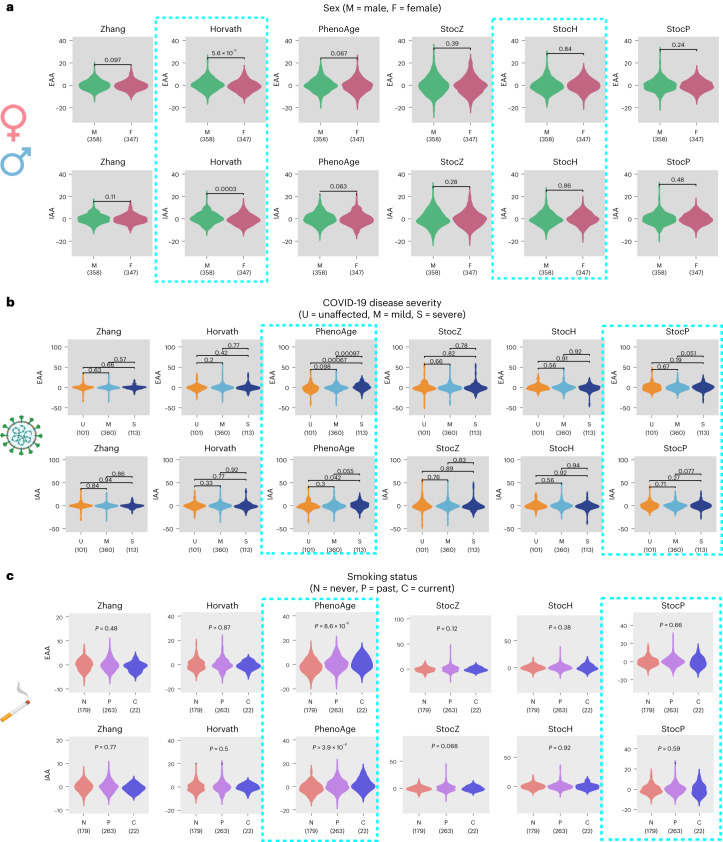
Age acceleration in males, severe COVID-19 cases and smokers is the result of nonstochastic processes. **a**, Violin plots for EAA and IAA (adjusted for 12 immune cell type fractions) for Zhang, Horvath and PhenoAge clocks, as well as their stochastic analogs. The *P* values derive from two-tailed Wilcoxon rank sum tests correlating the age acceleration measures to sex (male (M) and female (F)). Data are displayed for the TZH cohort but results are similar for all other cohorts. The number of samples in each violin plot is given below the violin. **b**, As in **a**, but evaluated in the DNAm dataset of Barturen et al., with EAA/IAA being correlated to COVID-19 disease severity, as indicated. **c**, As in **a**, but evaluated in the DNAm dataset of Tsaprouni et al., with EAA/IAA being correlated to smoking status. The *P* values derive from a linear regression test, with smoking status an ordinal variable (never smoker 0, ex-smoker 1 and smoker 2). Created with Biorender.com.

We next explored whether our stochastic clocks could be informative of biological age. We first focused on sex, since the increased epigenetic clock acceleration in males compared with females has been fairly well established, albeit only for Horvath’s clock^17^. Consistent with this, we observed that Horvath clock’s extrinsic and intrinsic age-acceleration measures (EAA and IAA) were positively correlated with male sex (Fig. 5a). This association was evident in 13 of the 16 whole blood cohorts (Extended Data Fig. 4a). In contrast, the stochastic analog (StocH) did not display significant age acceleration in males despite this clock being made up of exactly the same CpGs (Fig. 5a and Extended Data Fig. 4a). This suggests that age acceleration in males cannot be explained by an increase rate of stochastic change, but rather that it is driven by nonstochastic processes. Indeed, it is worth pointing out that of the 13 whole blood datasets where EAA was correlated with male sex, that in 12 of these, the corresponding IAA was either not significantly correlated or significantly less so (Extended Data Fig. 4a), indicating that subtle nonstochastic shifts in immune cell composition between males and females^42^ could be driving the association of Horvath’s EAA with sex.

Next, we focused on coronavirus disease 2019 (COVID-19) severity. Recent work by Cao et al. highlighted age acceleration (for both EAA and IAA) in severe COVID-19 cases compared with mild cases and unaffected individuals, albeit only for Levine’s PhenoAge clock^43^. This was a surprising result to us because severe COVID-19 infection is well associated with pronounced shifts in blood cell type composition^42,44^. Indeed, the application of EpiDISH to the same dataset to estimate fractions for 12 immune cell types^42^ revealed that PhenoAge’s EAA association with severe COVID-19 disease vanished when considering the IAA (Fig. 5b). In contrast, both EAA and IAA measures of the StocP clock did not correlate with COVID-19 disease severity (Fig. 5b). Thus, our analysis indicates that the reported age acceleration in severe COVID-19 cases is the result of changes in immune cell type composition, thus reflecting a nonstochastic process, consistent with the nonsignificant associations obtained with the StocP clock.

As a final example, we considered the case of smoking and obesity. As expected, the PhenoAge clock displayed significant age acceleration (both EAA and IAA) in smokers, although this was only seen in four of eight cohorts with available smoking information (Fig. 5c and Extended Data Fig. 4b). As with sex and COVID-19 disease severity, these associations vanished when considering the StocP clock (Fig. 5c and Extended Data Fig. 4b). Thus, in this case, although the association for PhenoAge clock persists upon adjusting for all 12 immune cell fractions, it is not present for the stochastic analog, suggesting that the age acceleration in smokers is driven by a nonstochastic process unrelated to shifts in immune cell composition. A similar pattern was also evident for body mass index (Extended Data Fig. 4c). In summary, these results indicate that reported age accelerations of Horvath- and PhenoAge clocks are probably the result of nonstochastic processes as opposed to an increase in the rate of stochastic DNAm change.

### Increased stochastic rate of change underpins mitotic age acceleration

**Fig. 6:**
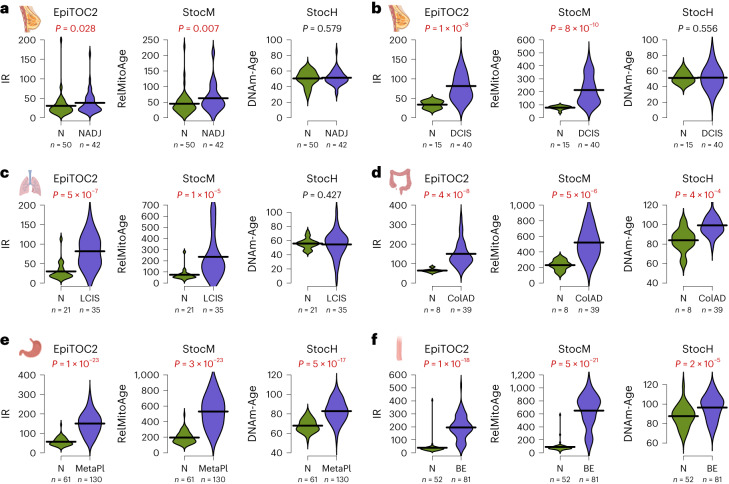
An increased rate of stochastic change describes mitotic age acceleration in precancer. **a**, Left: violin plots comparing the IR per year between normal breast from healthy women (N) and normal breast adjacent to breast cancer (NADJ), as estimated using the epigenetic mitotic clock EpiTOC2. The *P* values are from a one-tailed Wilcoxon test. The number of samples is given below violin. Middle and right: as left, but for the stochastic analog of EpiTOC2 and StocH. Samples between groups are age matched. **b**, As in **a** but for a DNAm dataset profiling normal breast tissue and breast DCIS. **c**, As in **a** but for a DNAm dataset profiling normal lung tissue and LCIS. **d**, As in **a** but for a DNAm dataset profiling normal colon tissue and colon adenoma (ColAD). **e**, As in **a** but for a DNAm dataset profiling normal gastric mucosa and gastric metaplasia (MetaPl). **f**, As in **a** but for a DNAm dataset profiling normal squamous esophagus and Barrett’s esophagus (BE). Created with Biorender.com.

While all previous examples illustrate how epigenetic age acceleration in blood requires nonstochasticity, we reasoned that an exception to this would be CpGs that track mitotic age^32,39,45^. Indeed, DNAm maintenance errors have long been hypothesized to accrue quasi-stochastically following cell division^46,47^, and given that the rate of stem cell division increases in precancerous and cancer conditions^45,48^, we thus reasoned that mitotic age acceleration in precancerous conditions would be detectable using a fully stochastic clock. We focused on 163 CpGs that make up a mitotic clock called EpiTOC2 (ref. ^39^) and, using the same strategy as for the other clocks, we built a fully stochastic version of it called StocM (Methods). We then assessed both EpiTOC2 and StocM in six independent DNAm datasets profiling normal and precancerous samples from solid tissues including breast, lung, colon, stomach and esophagus (Methods). Validating our hypothesis, StocM displayed clear mitotic age acceleration in all precancerous conditions, with a level of statistical significance very similar to that attained by EpiTOC2 itself (Fig. 6). As the data are from solid tissues, we also assessed the stochastic analog of the multitissue Horvath clock (StocH). In contrast to StocM, StocH did not display a consistent increased age acceleration in all datasets, and in those where there was age acceleration, the level of statistical significance was much lower compared with StocM (Fig. 6). This is consistent with the nonmitotic nature of most of Horvath clock CpGs^45^. Overall, these data support the view that the process by which DNAm changes accrue following stem cell division is a quasi-stochastic process and, consequently, that an increased rate of stem cell division in a tissue, as observed in precancer states, can be described as an increased rate of stochastic change.

## Discussion

Using 25 independent DNAm datasets encompassing over 22,000 whole-blood and sorted immune cell samples, we have here quantified how much of the predictive accuracy of epigenetic clocks could be driven by a stochastic process of cumulative DNAm change. This was done by focusing on the CpGs that make up three distinct clocks and building stochastic analogs from artificial cohorts generated through a stochastic process of DNAm change accrual. By considering three DNAm clocks (Zhang, Horvath and PhenoAge) that vary in their predictive accuracy of chronological and biological age, we have demonstrated that the more predictive a clock is of chronological age, the more this predictive accuracy could be driven by a pure stochastic process.

This insight has deep implications. It strongly suggests that processes that underlie biological aging are not the result of an increased rate of stochastic DNAm change, but rather the result of nonstochastic mechanisms^37^. Consistent with this, we find that several of the reported associations between epigenetic clock age-acceleration measures and phenotypes are driven by nonstochastic processes such as shifts in immune cell type composition^49^, a concrete example being the reported age acceleration with COVID-19 disease severity^43^. In the case of sex, Horvath’s clock still displayed an association after adjustment for immune cell counts but in most cohorts, this association disappeared or weakened. In all cohorts, no association was evident upon using the stochastic analog clock (StocH), suggesting that age acceleration in males is mainly the result of nonstochastic processes, including shifts in immune cell composition. In this context, it is worth noting that we recently demonstrated significant shifts in immune cell composition between males and females, including increased naive CD4^+^ and CD8^+^ T cell fractions in females^42^. Although linear adjustment for 12 immune cell counts, as done here, can address confounding by CTH, it is worth noting that a posteriori correction may not fully adjust for subtle changes in immune cell composition, specially if some of the original Horvath CpGs are capturing such changes^49^. In the case of smoking, cohorts that displayed significant extrinsic PhenoAge acceleration did so also for the IAA, but not when considering the StocP clock. Thus, biological age acceleration in smokers seems associated with nonstochastic processes that are unrelated to shifts in immune cell composition. This is not inconsistent with the high reproducibility of smoking-related DNAm signatures that reflect a reaction to smoking exposure, mapping to the nicotinic acetylcholine receptor and detoxification enzyme pathways^50–54^, an inherently nonstochastic process.

Our work also highlights the striking difference between traditional epigenetic clocks and those that track mitotic age. It is widely known that a substantial component of age-associated DNAm changes in mitotic tissues is associated with cell division^32,45–47,55–57^. If one restricts to the pool of CpGs that preferentially gain or lose DNAm following cell division (that is, PRC2-marked sites for hypermethylation), the pattern of DNAm changes appears to be stochastic^39^. Consequently, the increased stem cell division rate, which is thought to underpin cancer risk^48,58^, would be reflected by an increased stochastic rate of DNAm change at these sites. Consistent with this, building a stochastic analog of the EpiTOC2 mitotic clock resulted in a clock that displayed an increased mitotic age acceleration in precancer states, similar to EpiTOC2 itself, suggesting that the PRC2-marked CpGs that make up EpiTOC2 are already capturing an inherently stochastic process of DNAm change. Using the 12-module composition model of epigenetic clocks as recently derived by Levine et al.^59^, the ‘pink’ and ‘navy-blue’ modules that make up EpiTOC2 would reflect a stochastic process of DNAm accrual associated with in vivo and, to some extent, also in vitro cell division. In contrast to StocM/EpiTOC2, the StocH clock displayed highly inconsistent patterns depending on tissue type, in line with the nonmitotic nature of Horvath’s clock CpGs^45^. Thus, while epigenetic mitotic clocks appear to largely capture an underlying stochastic process of age-related DNAm changes linked with cell division, the stochastic component of traditional epigenetic clocks is distinct. As illustrated by the Zhang clock, the stochastic process underlying its high prediction of chronological age is different to the one underpinning mitotic clocks, consistent with Zhang clock CpGs being made up of entirely different Levine modules (‘green–yellow’ and ‘orange’ modules)^59^. It will be very interesting for future work to explore the underlying stochasticity of these modules in more detail. In this regard, we note that although the Levine CpG modules were identified using unsupervised clustering, correlations driving such modules can naturally emerge among CpGs changing stochastically with age if the underlying stochastic DNAm changes display a strong directional skew, as indeed observed for these specific modules^59^.

It is important to note that the stochastic clock analogs built here constitute epigenetic clocks in their own right, consistent with the recent works of Tarkhov et al.^33^ and Meyer et al.^34^. Indeed, according to Horvath and Raj^17^, any age predictor that can achieve *R* values >0.8 in an independent large dataset encompassing a broad age range (for example, 20–100 years) deserves the label ‘epigenetic clock’ and while, for instance, StocH achieved an average *R*-value of ~0.74 (*R*^2^ ~ 0.55) in the sorted immune cell datasets, the 95% confidence interval (±1.96 × s.d.) includes the value 0.8. Given that many of the datasets analyzed here displayed age ranges less than 80 years, this suggests that the stochastic clocks built here would meet the strict definition of epigenetic clock as proposed by Horvath and Raj. In relation to this, it is equally important to stress that our work, as well as those of Meyer and Tarkhov^33,34^, do not imply that stochastic processes are necessary for accurate epigenetic age prediction; all these studies converge on pointing toward stochastic processes only being sufficient. Moreover, it is plausible that complex, as yet unknown, deterministic processes could give rise to the seemingly random age-associated DNAm patterns that underpin the predictive accuracy of chronological age. Indeed, it is worth noting the underlying simplicity of the stochastic models considered here as well as those of Meyer and Tarkhov^33,34^, when there is a whole plethora of unmodeled factors that probably contribute to the dynamic DNA methylome. For instance, while current proposed models are cell autonomous and largely nonmechanistic, more detailed mechanistic models that also incorporate other omic data types at cell type resolution (for example, single-cell sequencing assay for transposase-accessible chromatin and single-cell RNA-Seq) may in future help shed further light on the mechanisms underlying epigenetic clocks and aging^60^. Besides this limitation, here we also did not explore stochasticity at the level of spatial correlative patterns in real data^33^. Our findings are however broadly in agreement with those of Tarkhov et al.^33^, which indicated that most of the age-associated DNAm patterns are spatially stochastic.

In summary, by using over 20,000 DNAm samples from 25 independent datasets, this work has rigorously and accurately quantified the stochastic component of epigenetic clocks, demonstrating that stochasticity on its own can explain a substantial fraction of an epigenetic clock’s accuracy in predicting chronological age, with this fraction increasing in line with a clock’s predictive ability. Conversely, and with the exception of mitotic age, this indicates that biological-age acceleration is driven by nonstochastic processes. As such, this work significantly deepens our understanding of epigenetic clocks.

## Methods

### Ethics

All DNAm datasets analyzed here have already been published elsewhere. We refer to the respective publications. For the TruD cohort, already published previously by us^42^, participants provided written informed consent for participation and publication.

### Statistics and reproducibility

From a statistics perspective, the design of this study involves deriving *R*^2^ values for a number of different linear age predictors (the epigenetic clocks), as applied to a number of DNAm datasets. These DNAm datasets were composed of sorted immune cell samples and whole blood (for the application of epigenetic age clocks: Horvath, Zhang, PhenoAge, StocH, StocZ and StocP) and solid tissues for the application of epigenetic mitotic clocks (epiTOC2 and StocM). As such, the sample sizes of each DNAm dataset are fixed by the original study, that is, no samples were excluded unless otherwise stated. However, large sample size was one key criterion for the selection of all these DNAm datasets. Overall, we analyzed 15–16 whole-blood DNAm datasets, encompassing over 22,000 samples in total; 9 sorted immune cell datasets encompassing over 2,500 samples and 6 normal precancer datasets, encompassing 574 samples. In more detail, the sample sizes were as follows: whole blood: Airway (*n* = 1,032), Barturen (*n* = 574), Flanagan (*n* = 184), Hannon1 (*n* = 636), Hannon2 (*n* = 665), Hannum (*n* = 656), HPT_450k (*n* = 418), Johansson (*n* = 729), Lehne (*n* = 2707), LiuMS (*n* = 279), LiuRA (*n* = 689), Tsaprouni (*n* = 464), TZH (*n* = 705), Ventham (*n* = 380), Zannas (*n* = 422) and TruD (*n* = 10,050); sorted immune cells: MESA-Monocytes (*n* = 1,202, with *n* = 54 for effect size estimation and *n* = 1,148 for validation), MESA-CD4T-Cells (*n* = 214), BP-Monocytes (*n* = 139), BP-Neutrophils (*n* = 139), BP-naiveCD4T-cells (*n* = 139), Paul-Monocytes (*n* = 104), Paul-Tcells (*n* = 98), Paul-Bcells (*n* = 100) and Tserel-CD8T-cells (*n* = 100); solid tissues: lung preinvasive (*n* = 56), breast preinvasive (*n* = 55), gastric metaplasia (*n* = 191), Barret’s esophagus and adenocarcinoma (*n* = 157), colon adenoma (*n* = 47) and normal breast Erlangen (*n* = 92).

The number of DNAm datasets analyzed is sufficiently large to ensure statistical significance when comparing the ratio or *R*^2^ values between clocks using a paired Wilcoxon rank sum test. As only publicly available DNAm datasets were analyzed, experiments were not randomized and investigators were not blinded to allocation during experiments and outcome assessment.

### DNAm datasets of sorted samples and whole blood

#### Sorted immune cell datasets

We obtained DNAm profiles of immune cell sorted samples from the following sources, all encompassing Illumina 450k DNAm technologies: from the Reynolds et al. (MESA study)^61^ we obtained 1,202 monocyte and 214 CD4^+^ T cell samples (Gene Expression Omnibus (GEO): GSE56581); from BLUEPRINT (BP)^41^, we obtained 139 monocyte, 139 naive CD4^+^ T cell and 139 neutrophil samples from 139 individuals; from Tserel et al.^62^, we obtained 100 CD8^+^ T cell samples (GEO: GSE59065); and from Paul et al.^63^, we obtained 49 CD4^+^ T cell, 50 B cell and 52 monocyte 450k samples (EGA: EGAS00001001598). We used the normalized DNAm datasets as processed and described in the respective publications. The sex distribution of samples for those cohorts, where this information is available, is as follows: sex (no. males, no. females): BP_Mono (59, 80), BP_Tcell (59, 80), BP_Neu (59, 80), Paul_Mono (34, 70), Paul_Tcell (30, 68), Paul_Bcell (32, 68) and Tserel_CD8T (48, 52). The age distribution, that is, age (mean ± s.d. (minimum–maximum)): MESA_Mono (60 ± 9 (44–83)), MESA_CD4T (59 ± 9 (45–79)), BP_Mono (58 ± 11 (23–75)), BP_Tcell (58 ± 11 (23–75)), BP_NEU (58 ± 11 (23–75)), Paul_Mono (35 ± 18 (4–75)), Paul_Tcell (35 ± 18 (4–75)), Paul_Bcell (34 ± 17,(4–73)) and Tserel_CD8T (52 ± 24 (22–84)).

#### Whole-blood datasets

We also analyzed a total of 16 whole-blood datasets. All whole-blood datasets used Illumina DNAm bead arrays (EPIC or 450k), and were processed and normalized exactly as described in our recent meta-analysis^42^. Sex information for cohorts where this information was available is as follows: sex (no. males, no. females): Airway (621, 411), Barturen (287, 287), Flanagan (0, 184), Hannon1 (377, 259), Hannon2 (480, 185), Hannum (318, 338), HPT_450k (120, 298), Johansson (341, 388), Lehne (1,838, 869), LiuMS (77, 202), LiuRA (197, 492), Tsaprouni (327, 137), TZH (358, 347), Ventham (196, 184) and Zannas (122, 300). Age distribution was as follows: age (mean ± s.d. (minimum–maximum)): Airway (42 ± 8 (26–59)), Barturen (67 ± 17 (19–103)), Flanagan (56 ± 9 (35–83)), Hannon1 (40 ± 15 (18–90)), Hannon2 (45 ± 13 (18–81)), Hannum (64 ± 15 (19–101)), HPT_450k (61 ± 8 (34–91)), Johansson (47 ± 21 (14–94)), Lehne (51 ± 10 (24–75)), LiuMS (41 ± 11 (16–66)), LiuRA (52 ± 12 (18–70)), Tsaprouni (55 ± 7 (38–67)), TZH (55 ± 10 (19–71)), Ventham (37 ± 14 (17–79)), Zannas (42 ± 13 (18–77)) and TruD (54 ± 14 (3–98)).

### Single-cell model of stochastic age-related DNAm change

Ignoring allele-specific DNAm, at any given CpG in any given cell DNAm is effectively binary (0 unmethylated and 1 methylated). Without loss of generality we assume that a given CpG starts out unmethylated, that is *X*(*t* = 0) = 0. One can then model the change in DNAm over time as a two-state Markov Chain process specified by the following 2 × 2 transition probability matrix *P*:

$$P=\left(\begin{array}{cc}(1-p) & p\\ q & (1-q)\end{array}\right).$$

In the above, *p* is the probability of switching from the *X* = *0* state to a fully methylated one *X* = *1*, while *q* is the probability of binary DNAm-loss ($X=1\to X=0)$. From this model, and using the fact that ${P}^{n+1}={P}^{n}P$, one can derive recurrence relations for the matrix entries of the transition matrix at time step *t* + 1 (ref. ^64^). For instance,

$${P}_{01}^{(t+1)}=p+(1-q-p){P}_{01}^{(t)}$$

with starting value ${P}_{01}^{(0)}=0$. To find the steady-state probability we can set ${P}_{01}^{(t+1)}={P}_{01}^{(t)}={\pi }_{1}$ in the above equation so it must satisfy ${\pi }_{1}=p/(q+p)$ and, consequently, ${\pi }_{0}=q/(q+p)$. Defining a new variable ${y}_{t}={P}_{01}^{(t)}-{\pi }_{1}$, one can then show that

$${y}_{t+1}=\left(1-p-q\right){y}_{t},$$

which can be solved to yield the solution ${y}_{t}={\left(1-p-q\right)}^{t}{y}_{0}$, or alternatively,

$${P}_{01}^{(t)}=\frac{p}{(q+p)}-\frac{p}{(q+p)}{\left(1-p-q\right)}^{t}.$$

Assuming *p* = *q*, then in the steady state $({t}\to\infty)$, the probability of finding the CpG methylated is exactly 0.5. It follows by the binomial theorem that over a cell population, the measured DNAm value would also be 0.5. Hence, the methylation and demethylation probabilities *p* and *q* can be tuned to any desired steady-state value. In practice we know that these probabilities of methylation change are very small $({p},q \sim {10}^{-5}\ll 1)$ (ref. ^39^). Thus, assuming that all cells start out unmethylated, the above model implies that the DNAm beta-value fraction increases linearly with time until it reaches a nonlinear regime close to the steady-state value. Specifically, assuming very small *p*,*q* so that we can approximate ${(1-p-q)}^{t}\approx 1-\left(p+q\right)t$, it follows that for a CpG that starts out unmethylated in one cell, the probability of finding it methylated at time *t*, is

$${p}_{X=1}^{\left(t\right)}={p}_{X=0}^{\left(0\right)}{P}_{01}^{\left(t\right)}+{p}_{X=1}^{\left(0\right)}{P}_{11}^{\left(t\right)}={P}_{01}^{\left(t\right)}\approx {pt}.$$

Thus, by the binomial theorem, the DNAm fraction in a cell population also increases linearly with time.

Importantly, for CpGs that start out unmethylated and that typically map to regions of relatively high CpG density, one can further assume that $q\ll p$. Conversely, for CpGs that start out methylated and that often map to low-CpG-dense regions, one can assume that $p\ll q$. Thus, the more realistic model is one where either *p* or *q* is vanishingly small and where, with sufficient time, DNAm values would approach 0 or 1, respectively. Indeed, this model would be consistent with the big DNAm differences (ΔDNAm >0.8) as observed in long-term cell cultures^65^. In human tissues and on timescales of a human life, however, initially unmethylated or methylated CpGs would rarely display such big DNAm differences, as indeed typically we only observe 5% DNAm changes (ΔDNAm ~0.05) over a period of 50 years. This means that for studies profiling DNAm in human tissues, CpG DNAm levels are far from their putative steady-state values, which justifies using a linear approximation of the above model. We describe this approximation below.

### Stochastic simulation model for a cell population

To specify the simulation model, we use the youngest (*n* = 43) and oldest (*n* = 11) monocyte samples from the MESA study (*n* = 1,202) (ref. ^61^) to define the directionality of age-related change of the 353 Horvath clock CpGs (or alternatively the 514 Zhang clock or 513 PhenoAge clock CpGs), as well as to infer their starting and end-state methylation values and effect sizes. The youngest samples were of age 44 years (*n* = 2) and age 45 years (*n* = 41), while the oldest samples were all older than 80 years (five of age 81 years, five of age 82 years and one of age 83 years). From the young and old samples separately, we computed the average DNAm level for each of the clock CpGs and estimated the effect size accordingly as the difference in average DNAm between old and young. The average DNAm over young samples defines the starting DNAm level of each CpG. Let EffSize_*c*_ denote the effect size of CpG *c*, with the sign of this effect size determining the directionality of DNAm change, which is kept constant throughout the simulation. Of note, although in a single cell the DNAm level at a given CpG could be dynamic, for example, a DNAm gain could be followed by a DNAm loss and vice versa, the probability of these events occurring at the same locus is relatively small: since our simulation model operates at the level of a cell population, it is thus very reasonable to assume that DNAm changes at a given locus occur in a unidirectional fashion. Thus, at each time step of the simulation, we randomly change a given CpG’s *c* DNAm value according to a probability given by

$${p}_{c}=1-{e}^{-\gamma \left|{\mathrm{EffSize}}_{c}\right|},$$

where *γ* is a parameter that controls the global probability of a DNAm change. For larger *γ* values, the probability of a CpG’s DNAm value changing approaches 1. For small *γ* values, the probability of a CpG’s DNAm value changing increases linearly with its observed effect size.

For each CpG that needs to be altered, we than randomly pick a stochastic deviate from a truncated normal distribution, that is from $${{\mathscr{N}}}_{+}(0,\sigma )$$ if the effect size has a positive sign, otherwise from a corresponding truncated negative normal distribution. Thus, our simulation model is also specified by the parameter *σ*, which controls the magnitude of DNAm change. However, because DNAm values are beta-distributed and hence heteroscedastic, adding normal deviates to such beta-values would not preserve the beta-valued nature of the DNAm data. Thus, before adding stochastic normal deviates, we first transform the DNAm beta-value $${\beta }_{c}^{(t)}$$ at the given iteration *t* into normal quantiles using the inverse of the normal cumulative distribution function, *iF*. Mathematically, $${x}_{c}^{(t)}={iF}({\beta }_{c}^{\left(t\right)})$$. Then $${x}_{c}^{(t+1)}={x}_{c}^{(t)}+$$$$\mathrm{sign}({\mathrm{EffSize}}_{c}){r}_{\pm }$$ where $${r}_{\pm }$$ is a random deviate drawn from $${{\mathscr{N}}}_{\pm }(0,\sigma )$$, depending on the sign of the effect size. Finally, we transform back to DNAm beta-values: $${\beta }_{c}^{(t+1)}=F({x}_{c}^{\left(t+1\right)})$$.

### Parameter estimation

To estimate the parameters $\left(\gamma ,\sigma \right)$, we run the simulation a number of time steps and compare the end-state DNAm values of the 353 CpGs (or 514/513 CpGs in the case of Zhang/PhenoAge CpGs) to the observed ones derived from the 11 oldest samples. To give the time step a concrete biological meaning, we equate a time step with one cell division, although we stress that this is not necessary and the actual DNAm changes in real data may be unrelated to cell division. Since blood turns over at the rate of approximately 35 divisions per year^39^, and since there are 37 years inbetween the median youngest age (45 years) and median oldest age (82 years), the total number of time steps in our simulation is 37 × 35 = 1,95. To find the optimal parameter values, we implemented a recursive process defining two-dimensional grids of increased resolution, running a total of 50 simulations per grid value pair. The MAE between simulated and observed end-state DNAm values over the clock CpGs was then used as the metric to find the optimal $\left(\gamma ,\sigma \right)$ values.

### Construction of the stochastic Horvath, Zhang and PhenoAge clocks (StocH, StocZ and StocP)

To construct a stochastic analog of Horvath’s clock, we next used the simulation model with inferred optimal parameter values to simulate an artificial cohort of samples. We generated five samples per age value, with ages ranging from 45 to 83 years of age, for a total of 195 samples. Each sample’s DNAm profile was generated de novo by running the simulation model starting out from a initial DNAm profile drawn randomly from the 43 youngest samples. Of note, this means that not all 195 samples start out from a different ground state. Nevertheless, this procedure allows us to generate as many independent artificial DNAm datasets as possible: for our purposes, we generated three separate cohorts of 195 samples each, to be used for training, model selection and testing^66^. Using the training set, we implemented an elastic net regression model (elastic net parameter alpha of 0.5) for variable lambda penalty parameter values (lambda varied from 0 to 1 in units of 0.001, so a total of 1,001 values) using the glmnet R package. Of note, for each CpG, DNAm values were standardized to mean zero and unit variance before running glmnet. This standardization is important as this significantly reduces the influence/bias of baseline DNAm levels that could vary between cell types, at least when assessing clocks in a correlative sense. It is also very important to note that since the simulation model induces age-related DNAm changes at all of the Horvath clock CpGs, that none of these is a false positive and hence that regularization is not really necessary. Indeed, the 1,001 elastic net models were evaluated in the model selection set, identifying lambda of 0 (zero penalty) as the optimal model. Finally, this optimal model was validated in the artificial test cohort. This elastic net clock model defines our StocH clock. The exact same procedure was followed for the 514 Zhang clock and 513 PhenoAge clock CpGs, resulting in stochastic clock models that we call ‘StocZ’ and ‘StocP’, respectively.

### Quantification of stochastic component of epigenetic aging

The StocH/StocZ/StocP clocks were applied to sorted immune cell and whole blood datasets to yield DNAm-based estimates of chronological age (DNAm-Age), which we compared to the known ages of the samples, using the MAE as well as the Pearson correlation coefficient (*R*-value) and associated *P* value. To quantify the stochastic component of Horvath’s clock, for each dataset we directly compared the *R*^2^ value from the StocH clock to the corresponding *R*^2^ value from Horvath’s clock^14^ by computing the ratio, that is, *R*^2^(StocH)/*R*^2^(Horvath). We often abbreviate this ratio with the term RR2. Similar ratios were computed for StocZ and Zhang’s clock as well as StocP and PhenoAge. The DNAm-Ages of the Horvath, Zhang and PhenoAge clocks were computed using our own scripts and verified using the methyclock R Bioconductor package^67^. On effectively all datasets, the RR2 values were <1. This strongly supports the interpretation of RR2 as the fraction of an epigenetic clock’s accuracy that can be attributed to a pure stochastic process of DNAm change. To understand this, we first note that both StocH and Horvath clocks are built from the same underlying CpGs, that they both use all 353 CpGs (that is, the estimated regression coefficients are all nonzero) and that they were trained from an elastic net regression model at the same alpha value of 0.5. The only difference between the two clocks is that the StocH clock was trained from simulated data built from a pure stochastic process of age-related DNAm accrual, while Horvath’s clock was built from multitissue real DNAm datasets (predominantly whole blood) representing a real molecular aging process. Since the *R*^2^ value describes the fraction of variation explained by a given model, the ratio of *R*^2^ values describes the fraction of the age variation explained by Horvath’s clock that can be attributed to a pure stochastic process. Identical arguments apply to StocZ and Zhang’s clock, as well as to StocP and PhenoAge clock.

Of note, taking the ratio of *R*^2^ values (that is, RR2) has the significant advantage that this automatically adjusts for any intrinsic study specific biases. For instance, Zhang’s clock was built from a large number of 450k DNAm datasets, including some that were also analyzed here, which may naturally lead to higher *R*^2^ values for StocZ and Zhang in those specific datasets. Thus, by taking the ratio of the corresponding *R*^2^ values (*R*^2^(StocZ)/*R*^2^(Zhang)) we automatically adjust for this potential bias.

### Insensitivity of StocH clock to ground-state DNAm values

To assess the dependency of the StocH clock to the cell type used in its construction (that is, monocytes), we used two different approaches. First, we built a reduced StocH clock by restricting the construction of the clock to Horvath CpGs that (1) displayed similar ground-state DNAm values in monocytes and CD4^+^ T cells (beta-value difference <0.1) and (2) same directionality of DNAm change with age in both cell types. This reduced StocH clock was also trained on the monocyte data. Hence, we reasoned that the reduced clock should yield better prediction measures in the CD4^+^ T cells compared with the full StocH clock because the former is based on CpGs that have the same ground-state DNAm values in monocytes and CD4^+^ T cells. The second approach was to apply the StocH clock to sorted immune cell datasets representing other blood cell types to directly compare prediction performance of monocytes with these other immune cell subtypes.

### Estimation of immune cell type fractions in whole blood and definition of EAA and IAA

In all whole blood cohorts, we used our 12 immune cell type DNAm reference matrix for either the Illumina 850k or 450k datasets^42^, to estimate corresponding cell type fractions. We did this with the EpiDISH Bioconductor R package^68,69^. Specifically, we ran the epidish function with ‘RPC’ as the method and maxit of 500. Subsequently, EAA of a clock was defined as the residuals of a linear regression of predicted DNAm-Age against chronological age. IAA of a clock was defined as the residuals of a linear regression of predicted DNAm-Age against chronological age and 11 of the 12 immune cell type fractions (because only 11 are independent).

### DNAm datasets of solid tissues representing normal and precancer states

#### Lung preinvasive dataset

This is an Illumina 450k DNAm dataset of lung tissue samples that we have previously published^53^. We used the normalized dataset from Teschendorff et al.^53^ encompassing 21 normal lung and 35 age-matched lung-carcinoma in situ (LCIS) samples, and 462,912 probes after quality control. Of these 35 LCIS samples, 22 progressed to an invasive lung cancer.

#### Breast preinvasive dataset

This is an Illumina 450k dataset of breast tissue samples from Johnson et al.^70^. Raw idat files were downloaded from GEO under accession number GSE66313 and processed with minfi. Probes with sample coverage <0.95 (sample coverage is defined as the fraction of samples with detected probes (that is, *P* < 0.05)) were discarded. The remaining unreliable values were assigned NA (not available) and imputed with impute.knn (imputation with k-nearest neighbors) (*k* = 5) (ref. ^71^). After BMIQ (beta-mixture quantile) normalization, we were left with 448,296 probes and 55 samples, encompassing 15 normal-adjacent breast tissue and 40 age-matched ductal carcinoma in situ (DCIS) samples, of which 13 were from women who later developed an invasive breast cancer.

#### Gastric metaplasia dataset

Raw idat files were downloaded from GEO (GSE103186) (ref. ^72^) and processed with minfi. Probes with over 99% coverage were kept and missing values imputed using the impute R package using impute.knn (*k* = 5). Subsequently, data was intra-array normalized with BMIQ, resulting in a final normalized data matrix over 482,975 CpGs and 191 samples, encompassing 61 normal gastric mucosas, 22 mild intestinal metaplasias and 108 metaplasias. Although age information was not provided, we used Horvath’s clock^14^ to confirm that normal and mild intestinal metaplasias were age matched. This is justified because Horvath’s clock is not a mitotic clock^39^ and displays a MAE of ±3 years (ref. ^14^).

#### Barrett’s esophagus and adenocarcinoma dataset

This Illumina 450k dataset^73^ is freely available from GEO under accession number GSE104707. Data were normalized as described by us previously^74^. The BMIQ-normalized dataset is defined over 384,999 probes and 157 samples, encompassing 52 normal squamous epithelial samples from the esophagus, 81 age-matched Barrett’s esophagus specimens and 24 esophageal adenocarcinomas.

#### Colon adenoma dataset^75^

Illumina 450k raw idat files were downloaded from ArrayExpress E-MTAB-6450 and processed with minfi. Only probes with 100% coverage were kept. Subsequent data were intra-array normalized with BMIQ, resulting in a normalized data matrix over 483,422 CpGs and 47 samples, encompassing 8 normal colon specimens and 39 age-matched colon adenomas. Although age information was not made publicly available, we imputed them using Horvath’s clock, confirming that normals and adenomas are age matched.

#### Normal breast Erlangen dataset

This Illumina 450k dataset is freely available from GEO under accession number GSE69914. Data were normalized as described by us previously^76^. The BMIQ-normalized dataset is defined over 485,512 probes and 397 samples, encompassing 50 normal breast samples from healthy women, 42 age-matched normal-adjacent samples and 305 invasive breast cancers.

### Construction of stochastic mitotic clock (StocM) and estimation of mitotic age

As a model of an epigenetic mitotic clock, we focused on EpiTOC2 (ref. ^39^), which is based on 163 CpGs that (1) are constitutively unmethylated across many different fetal tissue types and (2) map to within 200 bp of a transcription start site. These CpGs are strongly enriched for sites marked by the polycomb-repressive-complex-2 (PRC2) in human embryonic stem cells. EpiTOC2 was built from fitting an explicit stochastic model of DNAm transmission between cell generations to real DNAm data^39^. As such, this model can be viewed as semi-stochastic because it is still trained from real DNAm data representing a real aging process. Thus, in analogy to the previous epigenetic clocks, we followed the exact same procedure to build a fully stochastic version of EpiTOC2 that we call ‘StocM’. Of note, because StocM is built from the same 163 CpGs that define EpiTOC2, this clock is not aimed at predicting chronological age, but instead yields a relative estimate of mitotic age (RelMitoAge). On real DNAm datasets, we applied EpiTOC2 to yield age-adjusted estimates of the intrinsic rate of stem cell division (IR) of each sample^39^. Likewise, we applied StocM to yield relative estimates of mitotic age. As a benchmark we also applied StocH, since the original Horvath clock is a multitissue age predictor, thus making it applicable to solid tissues.

### Reporting summary

Further information on research design is available in the Nature Portfolio Reporting Summary linked to this article.

## Supplementary information

Supplementary Information Supplementary Figs. 1–4.

Reporting Summary

Supplementary software contains glmStocALL.Rd object files containing the stochastic clocks as R objects and an R script RunStochClocks.R illustrating how to apply them. Note: the glmStocALL.Rd file can only be opened in the R software environment.

## Figures and Tables

**Extended Data Fig. 10:**
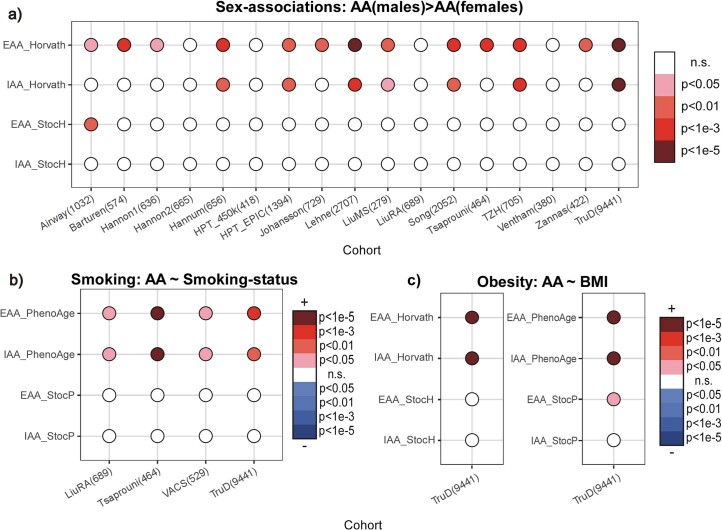
Stochastic clocks display reduced age-accelerations with sex, smoking and obesity. **a)** Balloon plot depicting associations of extrinsic and intrinsic age acceleration (EAA & IAA) with sex for the Horvath and StocH-clocks across 17 whole blood cohorts with available sex information. P-values derive from a one-tailed Wilcoxon rank sum test, with the alternative hypothesis being higher age-acceleration (AA) in males. **b)** Balloon plot depicting associations of extrinsic and intrinsic age acceleration (EAA & IAA) with smoking status for the PhenoAge and StocP-clocks across 4 whole blood cohorts with smoking information and where associations with smoking-status were found. P-value derives from a two-tailed correlation test. In the color-legend, + means higher AA in smokers. **c)** Balloon plot depicting associations of extrinsic and intrinsic age acceleration (EAA & IAA) with body-mass-index (BMI) for the Horvath/StocH and PhenoAge/StocP-clocks in the TruD cohort where an association of AA with BMI was found. P-value derives from a two-tailed correlation test. In the color-legend, + means higher AA in smokers.

## Data Availability

The following DNAm datasets are publicly available from GEO (www.ncbi.nlm.nih.gov/geo/) under accession numbers: GSE56581 (Reynolds et al. (MESA study), GSE59065 (Tserel), GSE40279 (Hannum), GSE42861 (LiuRA), GSE50660 (Tsaprouni), GSE106648 (LiuMS), GSE169156 (Song), GSE210255 (HPT-EPIC), GSE210254 (HPT-450k), GSE179325 (Barturen), GSE147740 (Airway), GSE117860 (VACS), GSE87648 (Ventham), GSE84727 (Hannon2), GSE80417 (Hannon1), GSE72680 (Zannas), GSE61151 (Flanagan/FBS), GSE87571 (Johansson), GSE55763 (Lehne), GSE66313 (breast preinvasive), GSE103186 (Gastric Metaplasia), GSE104707 (Barret’s esophagus and adenocarcinoma) and GSE69914 (normal breast Erlangen). The colon adenoma DNAm dataset was downloaded from ArrayExpress (https://www.ebi.ac.uk/biostudies/arrayexpress) under accession number E-MTAB-6450. The BLUEPRINT DNAm data of sorted monocytes, neutrophils and CD4^+^ T cells is available from European Genome Archive (EGA) under accession number EGAS00001001456. The DNAm data of sorted CD4^+^ T cells, B cells and monocytes is available from EGA (EGAS00001001598). The Illumina EPIC DNAm data for the TZH cohort can be viewed at National Omics Data Encyclopedia (NODE) under accession number OEP000260, or directly at https://www.biosino.org/node/project/detail/OEP000260, and accessed by submitting a request for data access. Data usage shall be in full compliance with the Regulations on Management of Human Genetic Resources in China. The lung preinvasive dataset is available upon request to the corresponding author. The TruD DNA methylation dataset is available upon request to TruDiagnostic (TD) Inc. (varun@trudiagnostic.com). To protect data privacy of the individuals represented in this cohort, individual applications will be reviewed by TD and in case TD is willing to share data, a data sharing agreement will be set up.

## Code availability

The stochastic clock predictors (StocH, StocZ and StocP) as well as an R script to estimate DNAm-Age and age accelerations according to these clocks are freely available from figshare at 10.6084/m9.figshare.24168483 or Supplementary Software. An R function implementing EpiTOC2 is freely available from https://github.com/aet21/EpiMitClocks.
