# Supplementary material for: Quantifying the stochastic component of epigenetic aging
Source: Nat Aging. 2024 May 9;4(6):886–901. doi: 10.1038/s43587-024-00600-8 (PMC11186785; doi:10.1038/s43587-024-00600-8)
Supplement: Supplementary file 1 — Supplementary Figs. 1–4. [file 43587_2024_600_MOESM1_ESM.pdf]

nature aging

Analysis https://doi.org/10.1038/s43587-024-00600-8

Quantifying the stochastic component of epigenetic aging

In the format provided by the authors and unedited

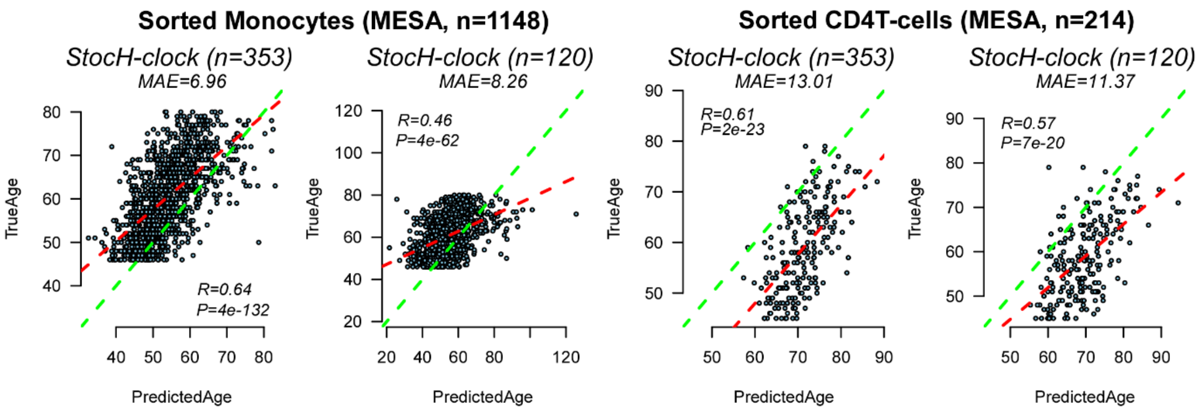

fig.S1: Comparison of the stochastic clocks trained on different subsets of Horvath clock CpGs in the independent sorted cells from MESA. Scatterplots of the true age vs predicted age for two stochastic clocks in the sorted monocyte and CD4+ T-cells of the MESA study as indicated. One stochastic clock was trained from all 353 Horvath clock CpGs, the other was trained on a subset of 120 CpGs that displayed similar DNAm levels in the youngest monocyte and CD4+ T-cell samples and that also displayed the same directionality of change with age in both immune cell-types. For each clock in each dataset, we provide the R-value, the corresponding nominal correlation-test two-tailed P-value and the median absolute error (MAE).

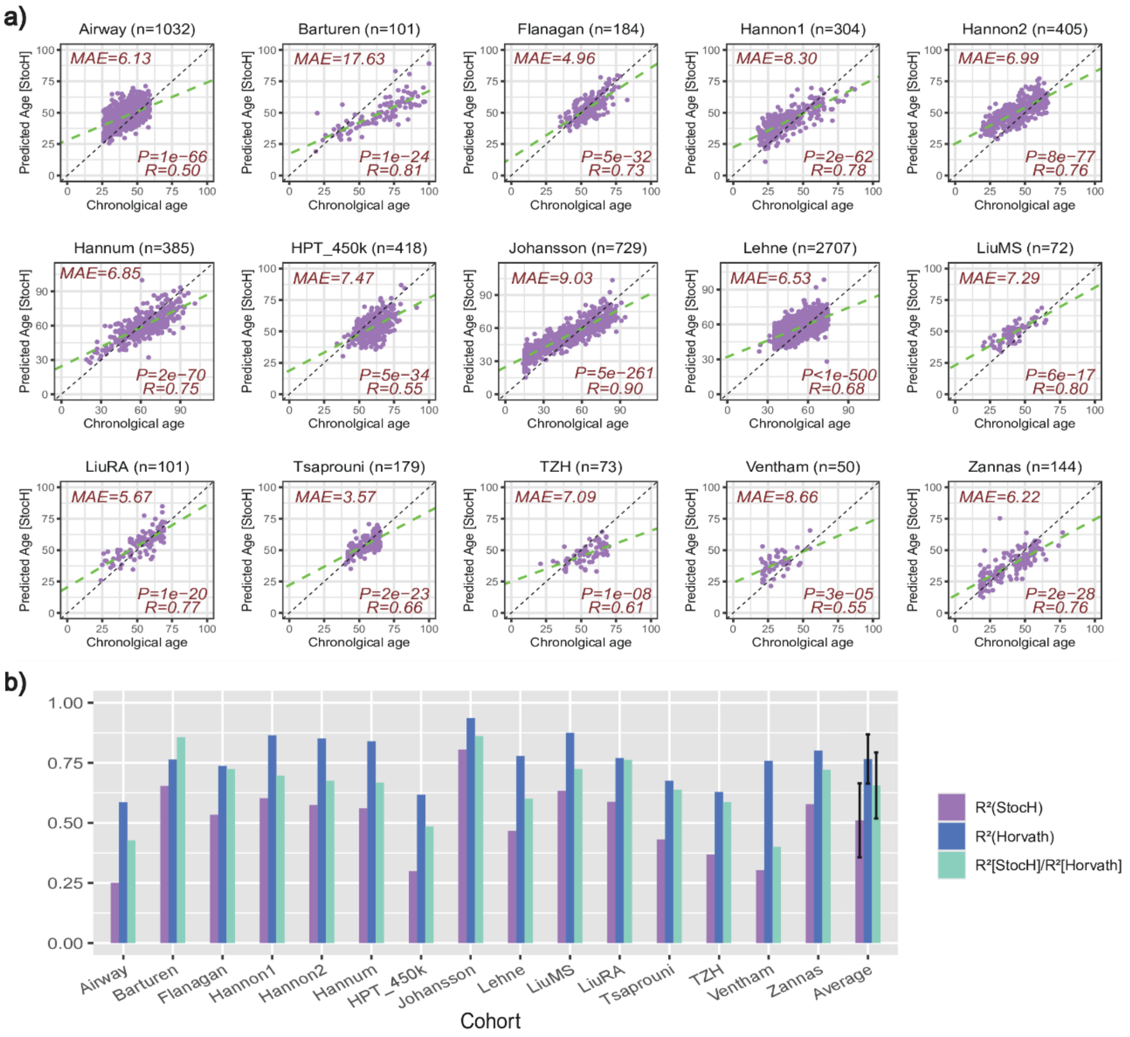

fig.S2: Quantification of the stochastic component of Horvath's clock in whole blood datasets, restricting to healthy samples only. a) Scatterplots of predicted age vs chronological (true) age for the stochastic Horvath clock in whole blood datasets, restricting to healthy samples only. Number of healthy samples in dataset is indicated at the top of the panel alongside the name of the cohort/study. In addition, we provide the median absolute error (MAE), R-value and corresponding nominal correlation-test two-tailed P-value. **b)** R^2 values for StocH, Horvath's clock and their ratio across all whole blood datasets (restricting only to healthy samples). Sample sizes (only including healthy samples) are: Airway (n = 1032), Barturen (n = 101), Flanagan (n = 184), Hannon1 (n = 304), Hannon2 (n = 405), Hannum (n = 385), HPT_450k (n = 418), Johansson (n = 729), Lehne (n =2707), LiuMS (n = 72), LiuRA (n = 101), Tsaprouni (n = 179), TZH (n = 73), Ventham (n = 50), Zannas (n = 144). The last set of bars displays the average and standard deviation over all whole blood datasets.

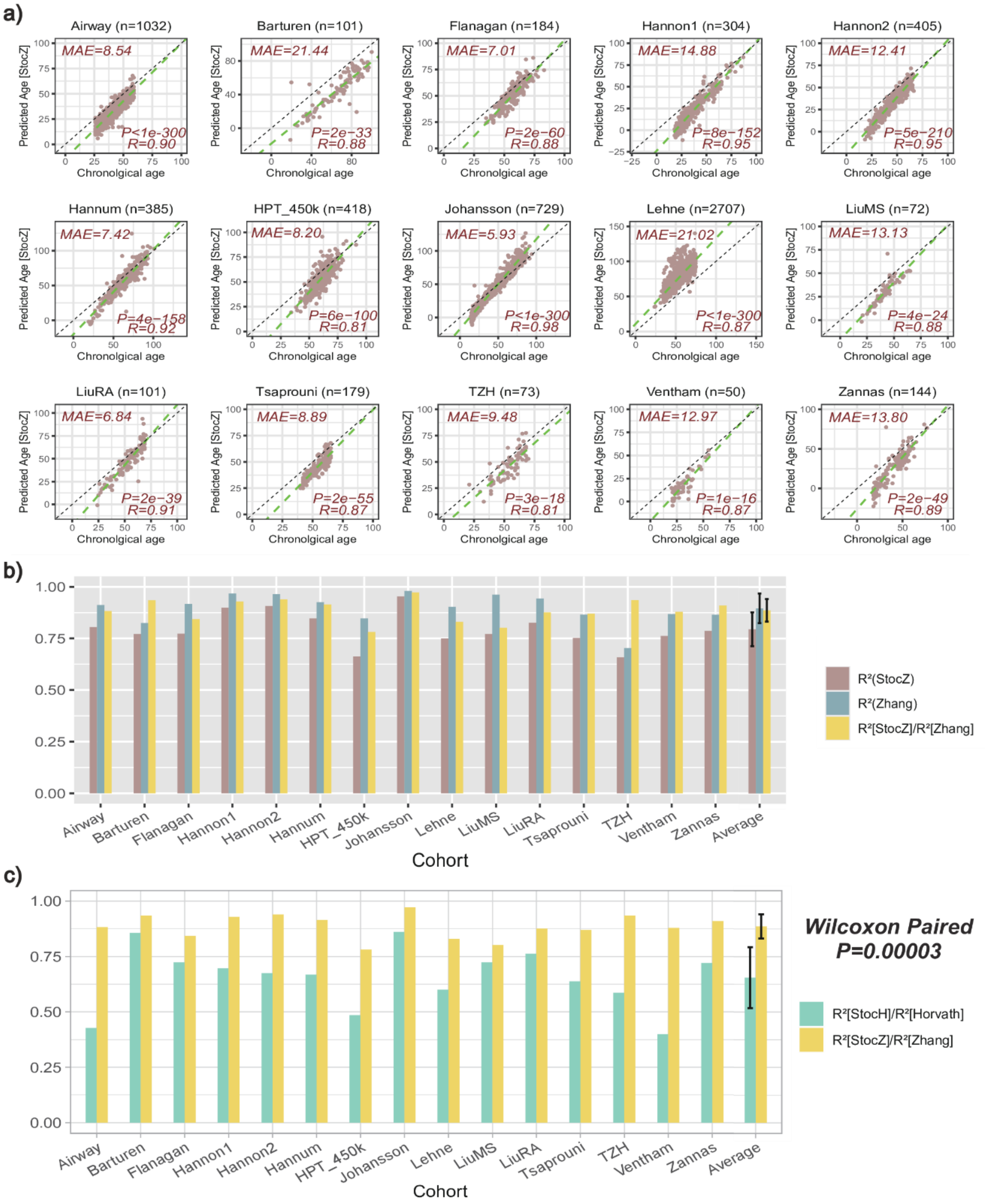

fig.S3: Quantification of the stochastic component of Zhang's clock in whole blood datasets, restricting to healthy samples only. a) Scatterplots of predicted age vs chronological (true) age for the stochastic Zhang clock in whole blood datasets, restricting to healthy samples only. Number of healthy samples in dataset is indicated at the top of the panel alongside the name of the cohort/study. In addition, we provide the median absolute error (MAE), R-value and corresponding nominal correlation-test two-tailed P-value. **b)** R^2 values for StocZ, Zhang's clock and their ratio across all whole blood datasets (restricting only to healthy samples). Sample sizes (only healthy samples) are: Airway (n = 1032), Barturen (n = 101), Flanagan (n = 184), Hannon1 (n = 304), Hannon2 (n = 405), Hannum (n = 385),

HPT_450k (n = 418), Johansson (n = 729), Lehne (n =2707), LiuMS (n = 72), LiuRA (n = 101), Tsaprouni (n = 179), TZH (n = 73), Ventham (n = 50), Zannas (n = 144). The last set of bars displays the average and standard deviation over all whole blood datasets. **c)** Barplot of ratio of R^2 values for the Horvath and Zhang CpGs across the whole blood datasets. Sample sizes as in b). The last set of bars displays the average and standard deviation over all whole blood datasets. P-value is from a one-tailed paired Wilcoxon rank sum test.

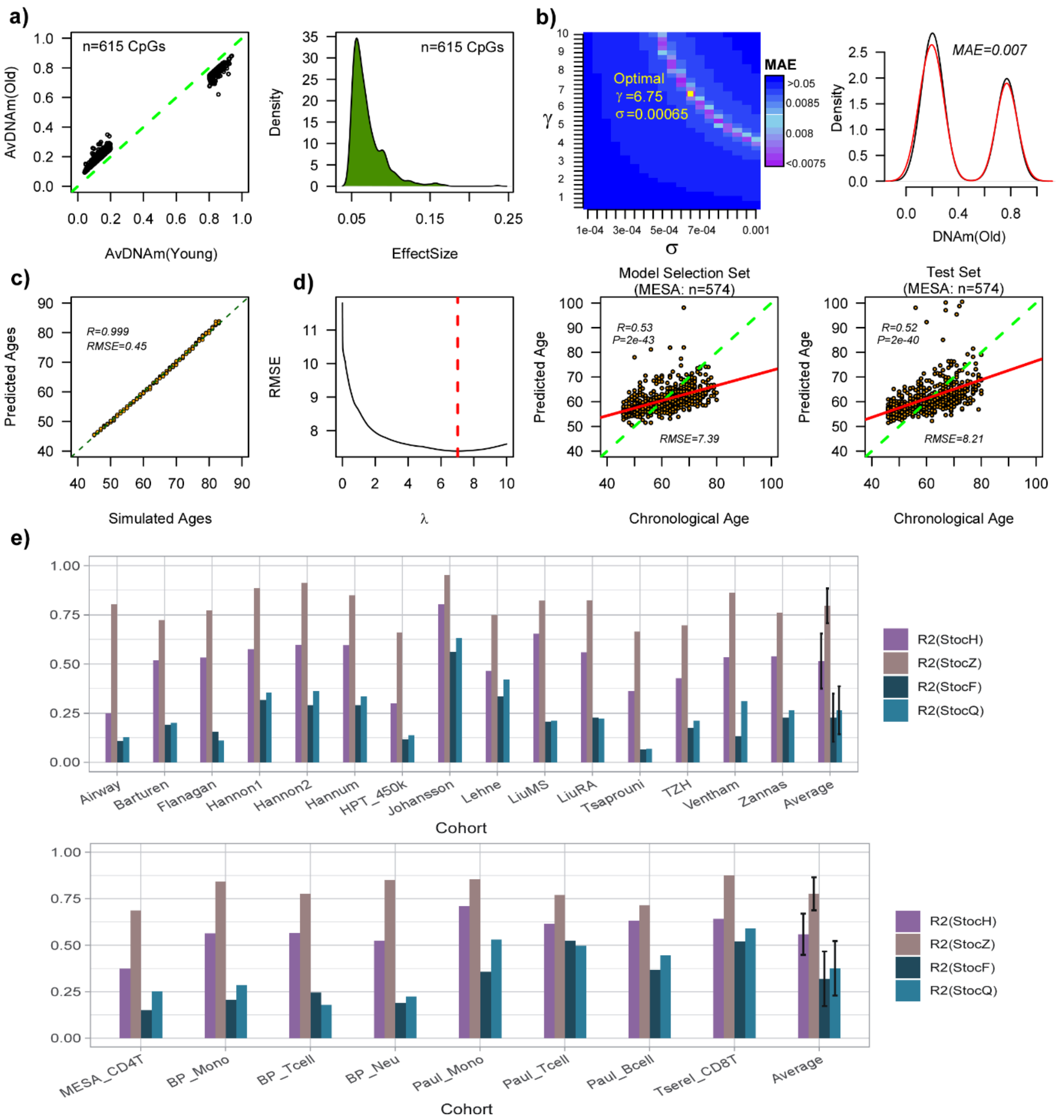

fig.S4: Construction and evaluation of fully stochastic and quasi-stochastic clocks. a) Scatterplot of average DNAm over young monocyte MESA samples vs old monocyte MESA samples for 615 age-associated CpGs (selected by comparing old and young MESA samples) with effect size > 0.05. Density plot shows the distribution of absolute effect sizes. **b)** Heatmap of median absolute error (MAE) for different choices of (γ , σ) in the simulation model. Right panel compares the simulated end-state DNAm distribution (i.e. old samples) of the 615 CpGs (red curve) to the observed one (black curve) for a simulation run at the optimal (γ , σ) values. **c)** Predicted age vs simulated (true) age of the optimal elastic net predictor (fully stochastic clock, StocF) constructed from simulation model at optimal (γ , σ) values, as evaluated in an independent simulated test set. R-value and RMSE (root-mean-square error) are given. **d)** Left: RMSE vs penalty parameter to select optimal penalty parameter λ for an elastic net predictor of age built from the same simulation model, but with RMSE now computed in 574 MESA samples, not used in the CpG selection. Middle: Predicted vs true age for the optimal model

(quasi-stochastic clock, StocQ) as assessed in the model selection set of 574 MESA samples. Right: Predicted vs true age for StocQ-clock in the other 574 MESA samples (also not used in CpG selection). R-value and RMSE values are given in middle and right panels. P-values in both panels derive from a nominal correlation-test (two-tailed). **e)** Barplot comparing R^2 values of four clocks (StocF, StocQ, StocH, StocZ) across whole blood and sorted immune cell datasets as shown. Sample sizes of whole blood datasets are: Airway (n = 1032), Barturen (n = 574), Flanagan (n = 184), Hannon1 (n = 636), Hannon2 (n = 665), Hannum (n = 656), HPT_450k (n = 418), Johansson (n = 729), Lehne (n =2707), LiuMS (n = 279), LiuRA (n = 689), Tsaprouni (n = 464), TZH (n = 705), Ventham (n = 380), Zannas (n = 422). Sample sizes of sorted immune cell datasets are: MESA_Mono (n = 1148), MESA_CD4T (n = 214), BP_Mono (n = 139), BP_Tcell (n = 139), BP_Neu (n =139), Paul_Mono (n = 104), Paul_Tcell (n = 98), Paul_Bcell (n = 100), Tserel_CD8T (n = 100). The last set of bars displays the average and standard deviation over all whole blood (upper panel) and sorted immune cell (lower panel) datasets.
