# Supplementary material for: Quantifying the stochastic component of epigenetic aging
Source: Nat Aging. 2024 May 9;4(6):886–901. doi: 10.1038/s43587-024-00600-8 (PMC11186785; doi:10.1038/s43587-024-00600-8)
Supplement: Supplementary file 2 — Reporting Summary [file 43587_2024_600_MOESM2_ESM.pdf]

nature portfolio

Corresponding author(s): Andrew E Teschendorff

Last updated by author(s): Jan 29, 2024

Reporting Summary

Nature Portfolio wishes to improve the reproducibility of the work that we publish. This form provides structure for consistency and transparency in reporting. For further information on Nature Portfolio policies, see our Editorial Policies and the Editorial Policy Checklist.

Statistics

For all statistical analyses, confirm that the following items are present in the figure legend, table legend, main text, or Methods section.

n/a	Confirmed	
☐	☒	The exact sample size (*n*) for each experimental group/condition, given as a discrete number and unit of measurement
☐	☒	A statement on whether measurements were taken from distinct samples or whether the same sample was measured repeatedly
☐	☒	The statistical test(s) used AND whether they are one- or two-sided<br>*Only common tests should be described solely by name; describe more complex techniques in the Methods section.*
☐	☒	A description of all covariates tested
☐	☒	A description of any assumptions or corrections, such as tests of normality and adjustment for multiple comparisons
☐	☒	A full description of the statistical parameters including central tendency (e.g. means) or other basic estimates (e.g. regression coefficient) AND variation (e.g. standard deviation) or associated estimates of uncertainty (e.g. confidence intervals)
☐	☒	For null hypothesis testing, the test statistic (e.g. F, t, r) with confidence intervals, effect sizes, degrees of freedom and P value noted<br>*Give P values as exact values whenever suitable.*
☐	☒	For Bayesian analysis, information on the choice of priors and Markov chain Monte Carlo settings
☒	☐	For hierarchical and complex designs, identification of the appropriate level for tests and full reporting of outcomes
☐	☒	Estimates of effect sizes (e.g. Cohen's *d*, Pearson's *r*), indicating how they were calculated

Our web collection on statistics for biologists contains articles on many of the points above.

Software and code

Policy information about availability of computer code

Data collection	No commercial code or software was used to download data. Data was downloaded manually from the websites hosting the data.
Data analysis	All analyses were performed using R-software version R_4.3.1 (https://cran.r-project.org) . The stochastic clock predictors (StocH, StocZ and StocP) as well as an R-script to estimate DNAm-Age and age-accelerations according to these clocks is freely available from figshare: https://doi.org/10.6084/m9.figshare.24168483 and Supplementary Software. The epigenetic clock estimates for Horvath, Zhang and PhenoAge clocks were derived using the methylclock Bioconductor R-package, version 1.6.0, available from https://bioconductor.org/packages/release/bioc/html/methylclock.html . An R-package EpiMitClocks, version 0.1.0, freely available from (https://github.com/aet21/EpiMitClocks) was used to obtain estimates of the mitotic clock. R-package glmnet version 4.1.7 was used to build elastic net regression models. EpiDISH version 2.16.0 was used to obtain cell-type fractions in blood. R-packages impute version 1.74.1 and minfi version 1.46.0 were used for normalization of DNAm datasets.

For manuscripts utilizing custom algorithms or software that are central to the research but not yet described in published literature, software must be made available to editors and reviewers. We strongly encourage code deposition in a community repository (e.g. GitHub). See the Nature Portfolio guidelines for submitting code & software for further information.

Data

Policy information about availability of data

All manuscripts must include a data availability statement. This statement should provide the following information, where applicable:

- Accession codes, unique identifiers, or web links for publicly available datasets
- A description of any restrictions on data availability
- For clinical datasets or third party data, please ensure that the statement adheres to our policy

The following DNAm datasets are publicly available from GEO (www.ncbi.nlm.nih.gov/geo/) under accession numbers: GSE56581 (Reynolds et al (MESA study), GSE59065 (Tserel), GSE40279 (Hannum), GSE42861 (LiuRA), GSE50660 (Tsaprouni), GSE106648 (LiuMS), GSE169156 (Song), GSE210255 (HPT-EPIC), GSE210254 (HPT-450k), GSE179325 (Barturen), GSE147740 (Airway), GSE117860 (VACS), GSE87648 (Ventham), GSE84727 (Hannon2), GSE80417 (Hannon1), GSE72680 (Zannas), GSE61151 (Flanagan/FBS), GSE87571 (Johansson), GSE55763 (Lehne), GSE66313 (Breast Preinvasive), GSE103186 (Gastric Metaplasia), GSE104707 (Barret's Esophagus & adenocarcinoma), GSE69914 (Normal Breast Erlangen). The colon adenoma DNAm dataset was downloaded from ArrayExpress (https://www.ebi.ac.uk/biostudies/arrayexpress) under accession number E-MTAB-6450. The BLUEPRINT DNAm data of sorted monocytes, neutrophils and CD4+ T-cells is available from EGA under accession number EGAS00001001456. The DNAm data of sorted CD4+ T-cells, B cells and monocytes is available from EGA: EGAS00001001598. The Illumina EPIC DNAm data for the TZH cohort can be viewed at NODE under accession number OEP000260, or directly at https://www.biosino.org/node/project/detail/OEP000260, and accessed by submitting a request for data-access. Data usage shall be in full compliance with the Regulations on Management of Human Genetic Resources in China. The Lung Preinvasive dataset is available upon request to the corresponding author. The TruD DNA methylation dataset is available upon request to TruDiagnostic (TD) Inc. (varun@trudiagnostic.com). In order to protect data privacy of the individuals represented in this cohort, individual applications will be reviewed by TD and in case TD is willing to share data, a data sharing agreement will be set up.

Field-specific reporting

Please select the one below that is the best fit for your research. If you are not sure, read the appropriate sections before making your selection.

☒ Life sciences ☐ Behavioural & social sciences ☐ Ecological, evolutionary & environmental sciences

For a reference copy of the document with all sections, see nature.com/documents/nr-reporting-summary-flat.pdf

Life sciences study design

All studies must disclose on these points even when the disclosure is negative.

Sample size	In this work, we meta-analyze approximately 25 DNAm whole blood and sorted immune-cell datasets, which are the largest available datasets. All these datasets together encompass over 20,000 samples. All the chosen datasets have the power to detect associations between epigenetic clocks and chronological age. Very small datasets containing less than 25 samples were never included in this study. As to the number of whole blood (15-16) and sorted immune-cell datasets (9) chosen these are sufficient to perform meta-analysis and detect significant differences in R2 values between clocks.
Data exclusions	No specific data or samples were excluded. When computing the R^2 values in the whole blood DNAm datasets, we did it both ways, including and not including samples from individuals with disease, which only had a very minor effect on results.
Replication	Our study-design involves a meta-analysis over 25 DNAm datasets, encompassing 20,000 samples. This constitutes ample replication and was done precisely to check if results generalize and are reproducible across independent datasets. All results were successfully reproduced.
Randomization	Randomization refers to the randomization in the design of a study that generates new data. Since here we only analyze publicly available datasets, randomization is not applicable.
Blinding	Stochastic epigenetic clocks were trained from simulation models built from the MESA study using only 54 of the youngest and oldest samples. ALL other datasets are blinded to this training data.

Reporting for specific materials, systems and methods

We require information from authors about some types of materials, experimental systems and methods used in many studies. Here, indicate whether each material, system or method listed is relevant to your study. If you are not sure if a list item applies to your research, read the appropriate section before selecting a response.

Materials & experimental systems

n/a	Involved in the study
☒	☐ Antibodies
☒	☐ Eukaryotic cell lines
☒	☐ Palaeontology and archaeology
☒	☐ Animals and other organisms
☒	☐ Human research participants
☒	☐ Clinical data
☒	☐ Dual use research of concern

Methods

n/a	Involved in the study
☒	☐ ChIP-seq
☒	☐ Flow cytometry
☒	☐ MRI-based neuroimaging
